# Supplementary material for: Benzo[1,2-d:4,5-d′]bis([1,2,3]thiadiazole) and Its Bromo Derivatives: Molecular Structure and Reactivity
Source: Int J Mol Sci. 2023 May 16;24(10):8835. doi: 10.3390/ijms24108835 (PMC10219027; doi:10.3390/ijms24108835)
Supplement: Supplementary file 1 [file ijms-24-08835-s001.zip › SuppInf_IJMS-12.pdf]

Supplementary Materials

Benzo[1,2-*d*:4,5-*d*']bis([1,2,3]thiadiazole) and its bromo derivatives: molecular structure and reactivity

Timofey N. Chmovzh [1,2], Daria A. Alekhina [1,3], Timofey A. Kudryashev [1,4], Rinat R. Aysin [5], Alexander A. Korlyukov [5] and Oleg A. Rakitin [1,*]

[1] N. D. Zelinsky Institute of Organic Chemistry, Russian Academy of Sciences, 119991 Moscow, Russia

[2] Nanotechnology Education and Research Center, South Ural State University, 454080 Chelyabinsk, Russia

[3] Higher Chemical College, Mendeleev University of Chemical Technology of Russia, Miusskaya Sq. 9, 125047 Moscow, Russia

[4] Department of Chemistry, Moscow State University, 119899 Moscow, Russia

[5] A. N. Nesmeyanov Institute of Organoelement Compounds, Russian Academy of Sciences, Moscow 119334, Russia

***** Correspondence: orakitin@ioc.ac.ru; Tel.: +7 499 135 5327

Table of contents:

1. **Calculations data**
2. **^{1}H and ^{13}C NMR spectra**
3. **Crystallographic data for 1- 3**

1. Calculations data

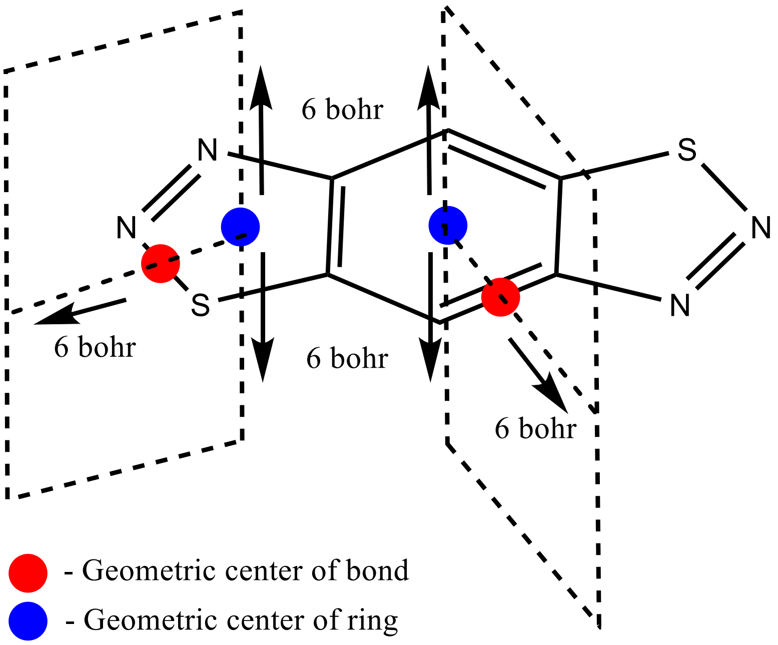

Scheme S1. The integration grid (dash line) scheme for IRCS evaluation.

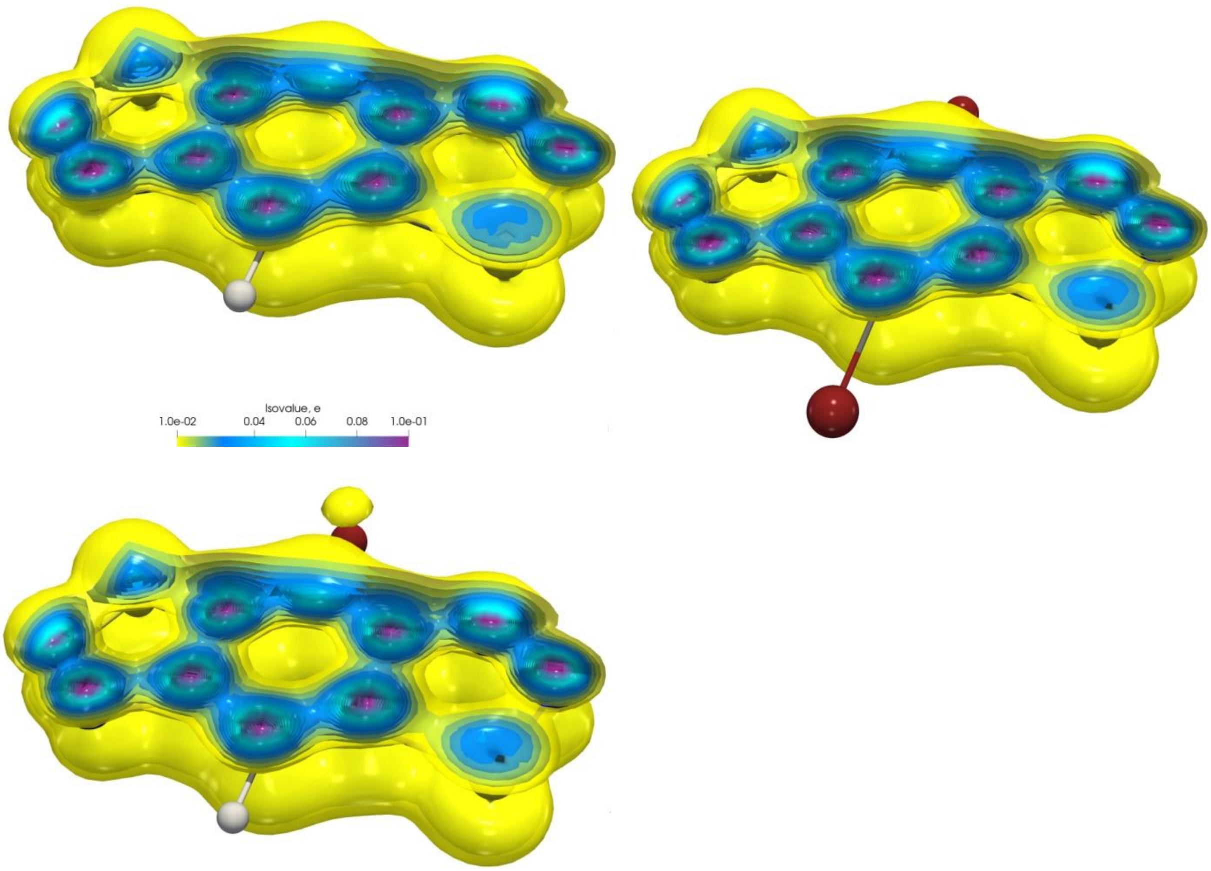

Figure S1. Multi-isosurface of π-EDDB$_H$ for **1-3**.

Table S1. The molecular π-orbitals for **1-3.**

**1**	**2**	**3**
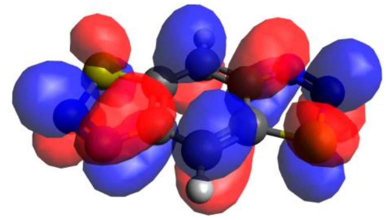 **LUMO**	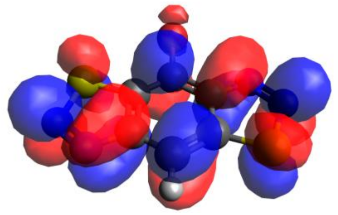 **LUMO**	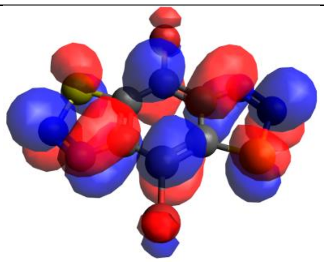 **LUMO**
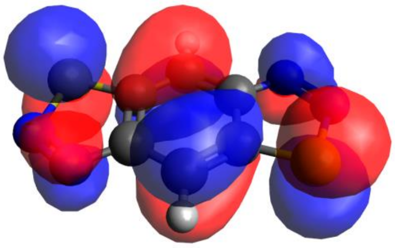 **HOMO**	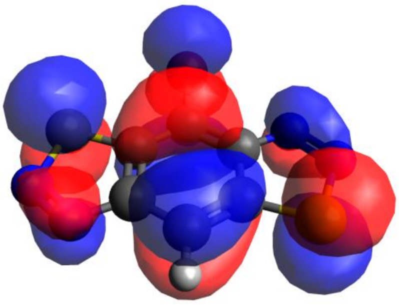 **HOMO**	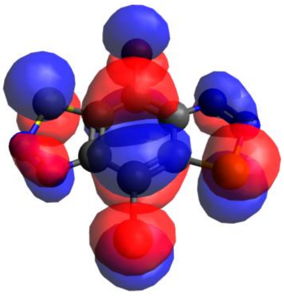 **HOMO**
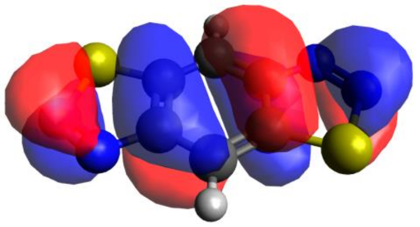 **HOMO-1**	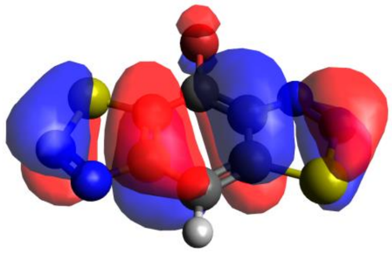 **HOMO-1**	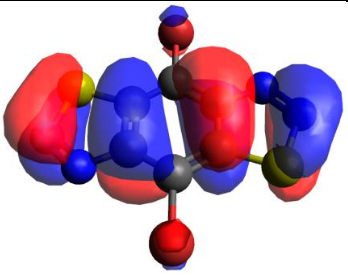 **HOMO-1**
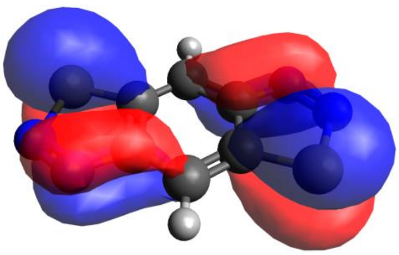 **HOMO-2**	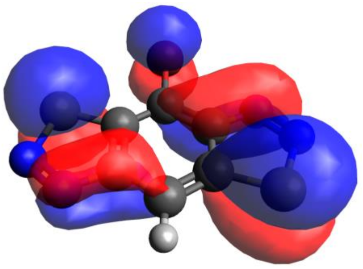 **HOMO-2**	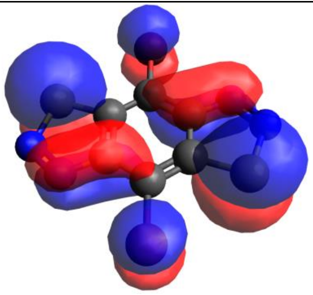 **HOMO-3**
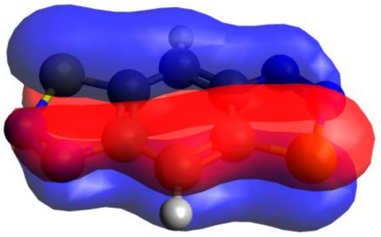 **HOMO-5**	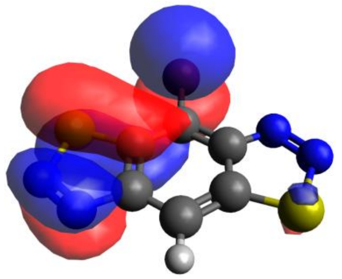 **HOMO-4**	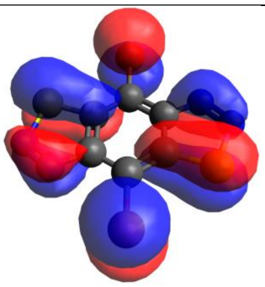 **HOMO-4**

1	2	3
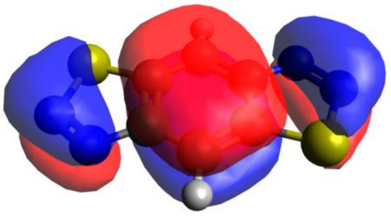 HOMO-6	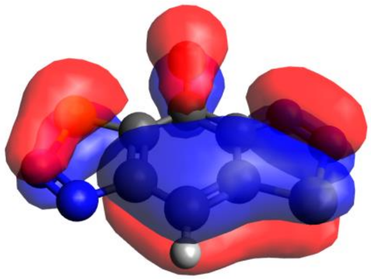 HOMO-8	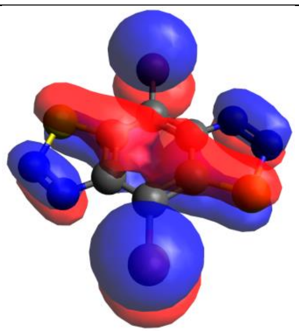 HOMO-5
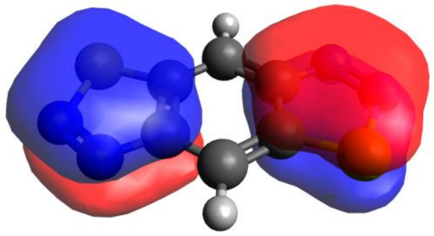 HOMO-11	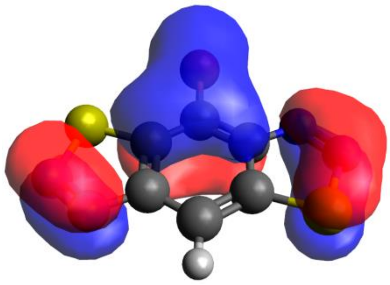 HOMO-9	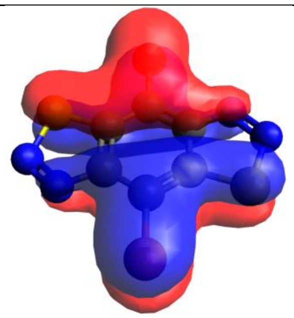 HOMO-10
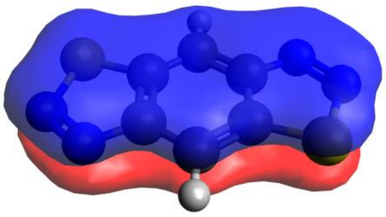 HOMO-13	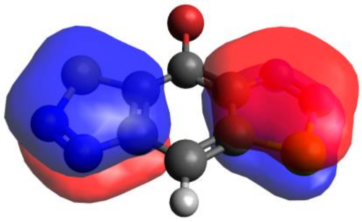 HOMO-14	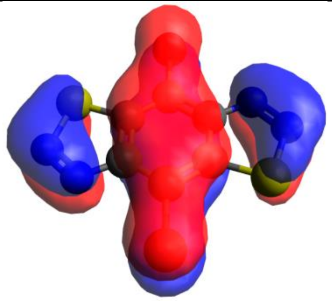 HOMO-11
	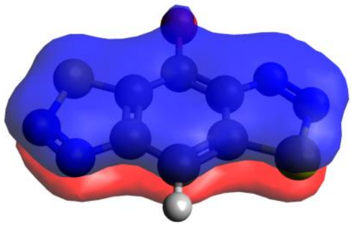 HOMO-16	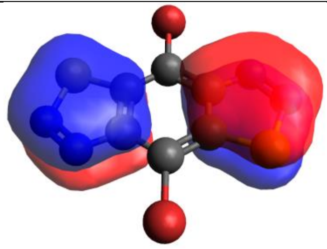 HOMO-16
		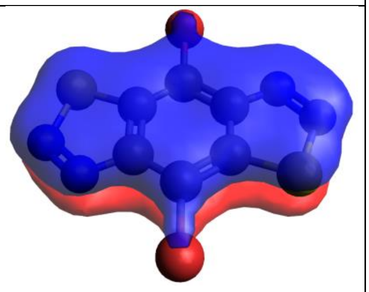 HOMO-18

Table S2. The GIMIC and EDDB results at the MP2/cc-pVTZ level for aromaticity evaluation in **BBT** in comparison to thiadiazoles and benzothiadiazoles.

MP2/<br>cc-pvtz	**BBT**	**isoBTD**	**BTD**		
	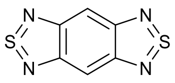	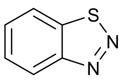	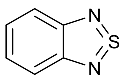	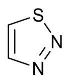	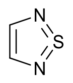
π-EDDB (5mr), ē	4.36 (72%)	3.45 (57%)	4.36 (73 %)	3.84 (64%)	3.81 (63%)
π-EDDB (6mr), ē	4.68 (78%)	4.11 (69%)	4.08 (68%)	–	–
π-EDDB overall, ē	10.30 (73%)	6.10 (61%)	6.89 (69%)	3.84 (64%)	3.81 (64%)
IRCS (5mr), nA/T	16.3	12.5	15.9	15.2	13.3
IRCS (6mr) , nA/T	16.3	13.7	12.1	–	–

Table S3. Calculated E_{tot} (bt-STEOM-CCSD) and ZPVE (TPSS-D4) value for the EA estimation.

	Vertical EA, eV	Neutral		Anion radical		Adiabatic EA $= -\Delta E$(attach), eV	
		E_{tot}, a.u.	ZPVE, a.u.	E_{tot}, a.u.	ZPVE, a.u.	With ZPVE correction	Without ZPVE correction
**1**	1.091	-1243.363822	0.077113	-1243.401352	0.074634	1.089	1.021
**2**	1.345	-3815.45658	0.06689235	-3815.502069	0.06457437	1.301	1.238
**3**	1.59	-6387.548802	0.056661	-6387.601969	0.05450211	1.506	1.447
**BBT**	1.792	-1243.382522	0.078261	-1243.450673	0.076502	1.902	1.855

Optimized *xyz*-Cartesian coordinates at MP2(fc)/cc-pVTZ level

14

1 E_{tot}= -1241.305265 a.u.

C	0.654811045	-1.212498088	-0.000444000
C	-0.775865055	-1.147957080	0.000550000
C	-0.654811045	1.212498088	0.000439000
C	0.775865055	1.147957080	-0.000559000
N	1.154602080	-2.491109179	-0.000773000
N	-1.154602080	2.491109179	0.000775000
N	0.245423018	-3.407998240	-0.000088000
N	-0.245423018	3.407997240	0.000171000
S	-1.345904095	-2.760667195	0.000932000
S	1.345904095	2.760667195	-0.001011000
C	1.451440105	-0.066194005	-0.001014000
H	2.529327180	-0.150339011	-0.001771000
C	-1.451440105	0.066194005	0.001007000
H	-2.529327180	0.150338011	0.001750000

14

1 anion radical E_{tot}= -1241.336227 a.u.

C	-0.799976000	1.133352000	0.000034000
C	0.636414000	1.247933000	0.000010000
C	0.799976000	-1.133352000	0.000034000
C	-0.636414000	-1.247933000	0.000010000
N	-1.439975000	2.319085000	0.000016000
N	1.439975000	-2.319085000	0.000016000
N	-0.726253000	3.370504000	0.000319000
N	0.726253000	-3.370504000	0.000319000
S	0.982863000	2.919149000	-0.000154000
S	-0.982863000	-2.919149000	-0.000154000
C	-1.439975000	-0.120911000	-0.000011000
H	-2.519946000	-0.169059000	-0.000074000
C	1.439975000	0.120911000	-0.000011000
H	2.519946000	0.169059000	-0.000074000

14

2 E_{tot}= -3813.169449 a.u.

C	0.649041000	-1.209971000	-0.000437000
C	-0.778838000	-1.145334000	0.000545000
C	-0.662654000	1.228326000	0.000455000
C	0.767819000	1.148013000	-0.000563000
N	1.141702000	-2.492360000	-0.000751000
N	-1.154139000	2.506031000	0.000790000
N	0.225175000	-3.400859000	0.000136000
N	-0.237388000	3.415690000	0.000358000
S	-1.361471000	-2.749869000	0.000770000
S	1.345693000	2.758473000	-0.001169000
C	1.445892000	-0.064971000	-0.001023000
H	2.522874000	-0.147048000	-0.001815000
C	-1.450149000	0.070857000	0.001008000
Br	-3.309785000	0.149861000	0.002249000

14

2 anion radical E_{tot}= -3813.21678 a.u.

C	0.642030000	-1.228576000	-0.000436000
C	-0.798276000	-1.154104000	0.000562000
C	-0.654047000	1.242540000	0.000443000
C	0.782187000	1.155457000	-0.000566000
N	1.115767000	-2.485004000	-0.000763000
N	-1.127612000	2.500143000	0.000763000
N	0.267382000	-3.430006000	-0.000101000
N	-0.276635000	3.437924000	0.000199000
S	-1.362496000	-2.757393000	0.000952000
S	1.347252000	2.766123000	-0.000995000
C	1.436266000	-0.066800000	-0.001011000
H	2.512511000	-0.154568000	-0.001770000
C	-1.433743000	0.066927000	0.000992000
Br	-3.306814000	0.174174000	0.002285000

14

3 E_{tot}= -6385.037866 a.u.

C	0.652174000	-1.224690000	-0.000447000
C	-0.776236000	-1.144996000	0.000550000
C	-0.663189000	1.225729000	0.000449000
C	0.765203000	1.146079000	-0.000557000
N	1.135939000	-2.505197000	-0.000769000
N	-1.147099000	2.506390000	0.000774000
N	0.210623000	-3.408316000	-0.000042000
N	-0.221398000	3.409349000	0.000157000
S	-1.367480000	-2.747012000	0.000928000
S	1.356159000	2.748068000	-0.001026000
C	1.440595000	-0.068464000	-0.001017000
C	-1.451793000	0.069305000	0.001012000
Br	-3.309939000	0.145826000	0.002299000
Br	3.298848000	-0.144573000	-0.002330000

14

3 anion radical E_{tot}= -6385.096184 a.u.

C	0.644534000	-1.241290000	-0.000443000
C	-0.793350000	-1.153671000	0.000564000
C	-0.655621000	1.242364000	0.000438000
C	0.782266000	1.154741000	-0.000568000
N	1.110620000	-2.497650000	-0.000752000
N	-1.121709000	2.498719000	0.000751000
N	0.252417000	-3.430431000	-0.000136000
N	-0.263488000	3.431502000	0.000142000
S	-1.364666000	-2.755404000	0.000981000
S	1.353572000	2.756479000	-0.000988000
C	1.422931000	-0.067115000	-0.001002000
C	-1.434015000	0.068186000	0.000998000
Br	-3.304474000	0.166592000	0.002306000
Br	3.293389000	-0.165522000	-0.002310000

14

4 E_{tot}= -1241.464940 a.u.

C	0.728099051	-1.182571082	0.000008000
C	-0.728099051	-1.182571082	0.000008000
C	-0.728098051	1.182563087	0.000005000
C	0.728098051	1.182563087	0.000005000
S	0.000000000	3.453772247	0.000034000
S	0.000000000	-3.453780243	0.000016000
N	-1.269658089	-2.438701173	0.000005000
N	1.269658089	-2.438701173	0.000005000
N	1.269657089	2.438694172	-0.000006000
N	-1.269657089	2.438694172	-0.000006000
C	-1.469381103	-0.000004000	0.000005000
H	-2.550365182	-0.000003000	0.000000000
C	1.469381103	-0.000004000	0.000005000
H	2.550365182	-0.000003000	0.000000000

14

4 anion radical E_{tot}= -1241.506221 a.u.

C	0.000001000	1.198147000	0.729743000
C	0.000001000	1.198147000	-0.729743000
C	0.000001000	-1.198148000	-0.729744000
C	0.000001000	-1.198148000	0.729744000
S	-0.000003000	-3.484333000	0.000000000
S	-0.000002000	3.484333000	0.000000000
N	0.000001000	2.434124000	-1.264885000
N	0.000001000	2.434124000	1.264885000
N	0.000003000	-2.434125000	1.264885000
N	0.000003000	-2.434125000	-1.264885000
C	0.000001000	0.000000000	-1.454508000
H	0.000002000	0.000000000	-2.535791000
C	0.000001000	0.000000000	1.454508000
H	0.000002000	0.000000000	2.535791000

12

5 E_{tot}= -1275.476017 a.u.

C	0.732144000	-1.105520000	-0.000038000
C	-0.732144000	-1.105520000	-0.000038000
C	-0.732145000	1.105520000	-0.000058000
C	0.732145000	1.105520000	-0.000058000
S	0.000000000	3.358836000	-0.000550000
S	0.000000000	-3.358837000	-0.000177000
N	-1.277084000	-2.352624000	0.000103000
N	1.277084000	-2.352624000	0.000103000
N	1.277084000	2.352624000	0.000377000
N	-1.277084000	2.352624000	0.000377000
N	-1.484682000	0.000000000	-0.000022000
N	1.484682000	0.000000000	-0.000022000

12

5 anion radical E_{tot}= -1275.537761 a.u.

C	0.729216000	-1.110542000	-0.000047000
C	-0.729099000	-1.110545000	-0.000048000
C	-0.729099000	1.110545000	-0.000105000
C	0.729217000	1.110542000	-0.000105000
S	-0.000096000	3.373230000	-0.000151000
S	-0.000096000	-3.373230000	0.000013000
N	-1.263854000	-2.332618000	0.000052000
N	1.263776000	-2.332650000	0.000053000
N	1.263776000	2.332650000	0.000196000
N	-1.263854000	2.332617000	0.000198000
N	-1.470989000	0.000000000	-0.000028000
N	1.471100000	0.000000000	-0.000027000

2. ^{1}H and ^{13}C NMR spectra

4-(Benzo[1,2-d:4,5-d']bis([1,2,3]thiadiazole)-4-yl)morpholine (4a)

^{1}H NMR (300 MHz)

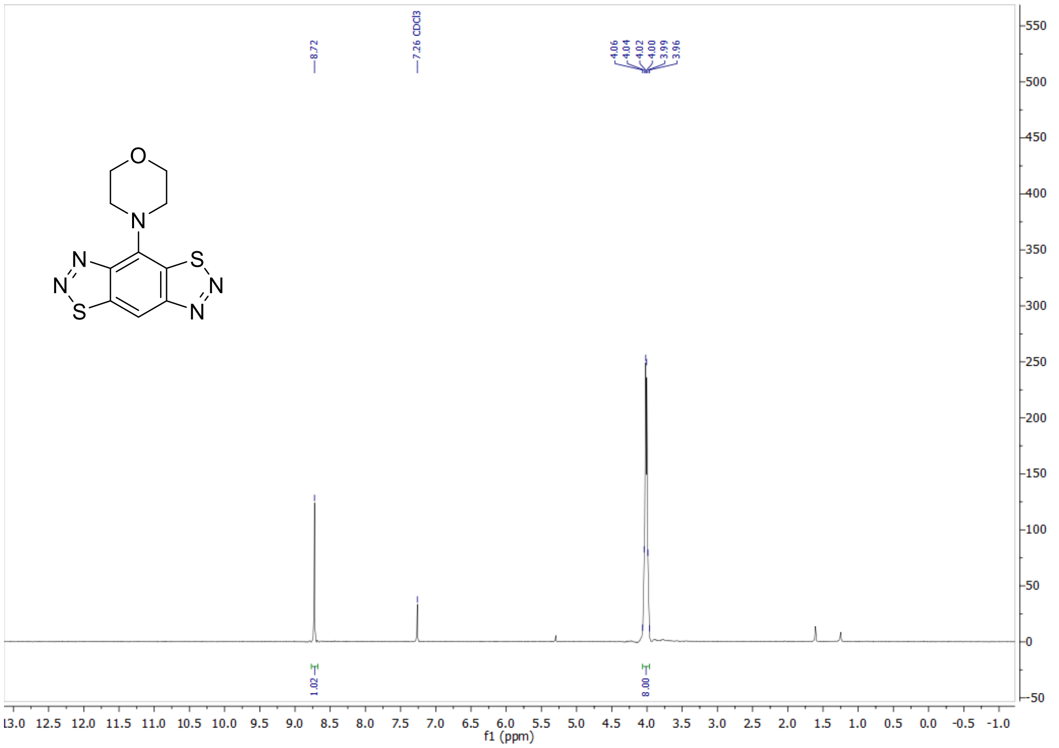

^{13}C NMR(75 MHz)

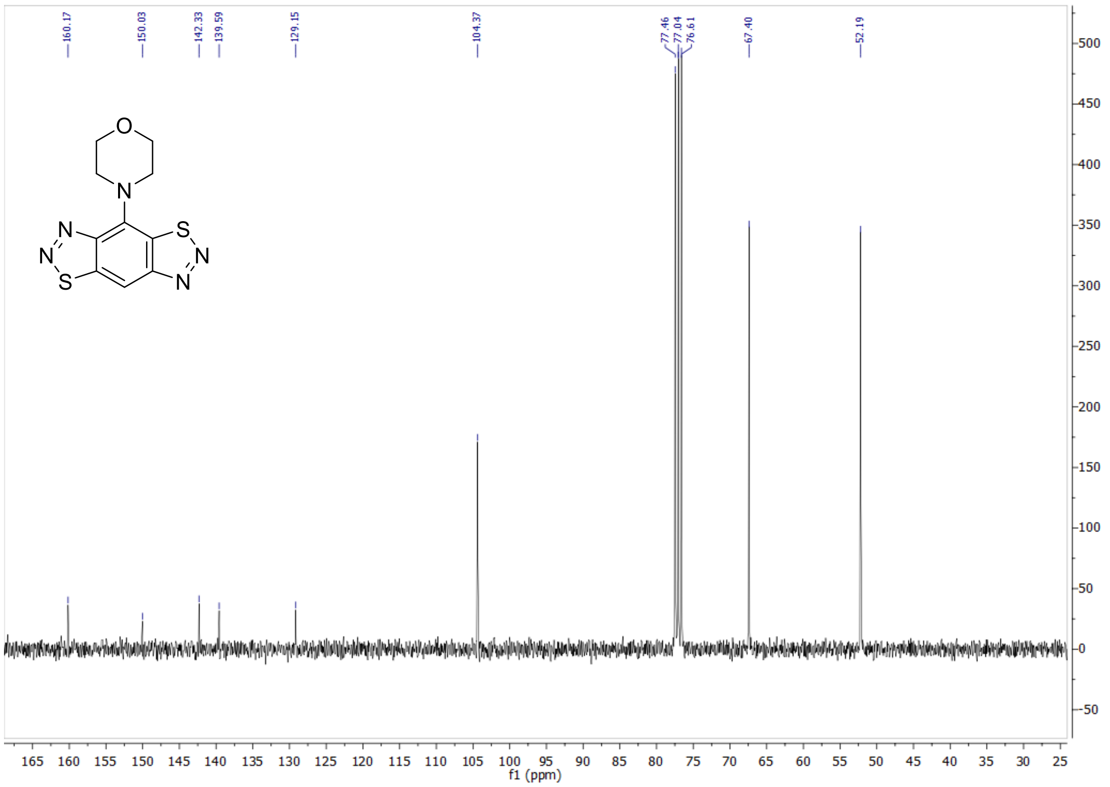

4-(Piperidin-1-yl)benzo[1,2-d:4,5-d']bis([1,2,3]thiadiazole) 4b

^{1}H NMR (300 MHz)

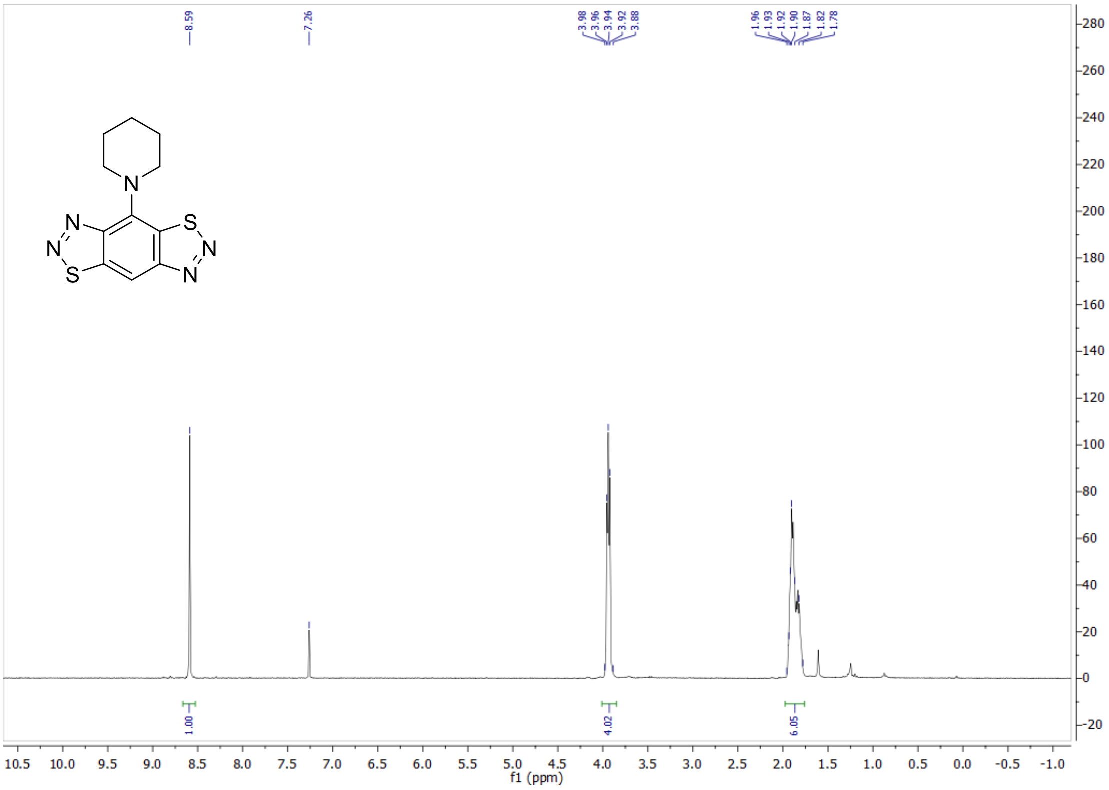

^{13}C NMR(75 MHz)

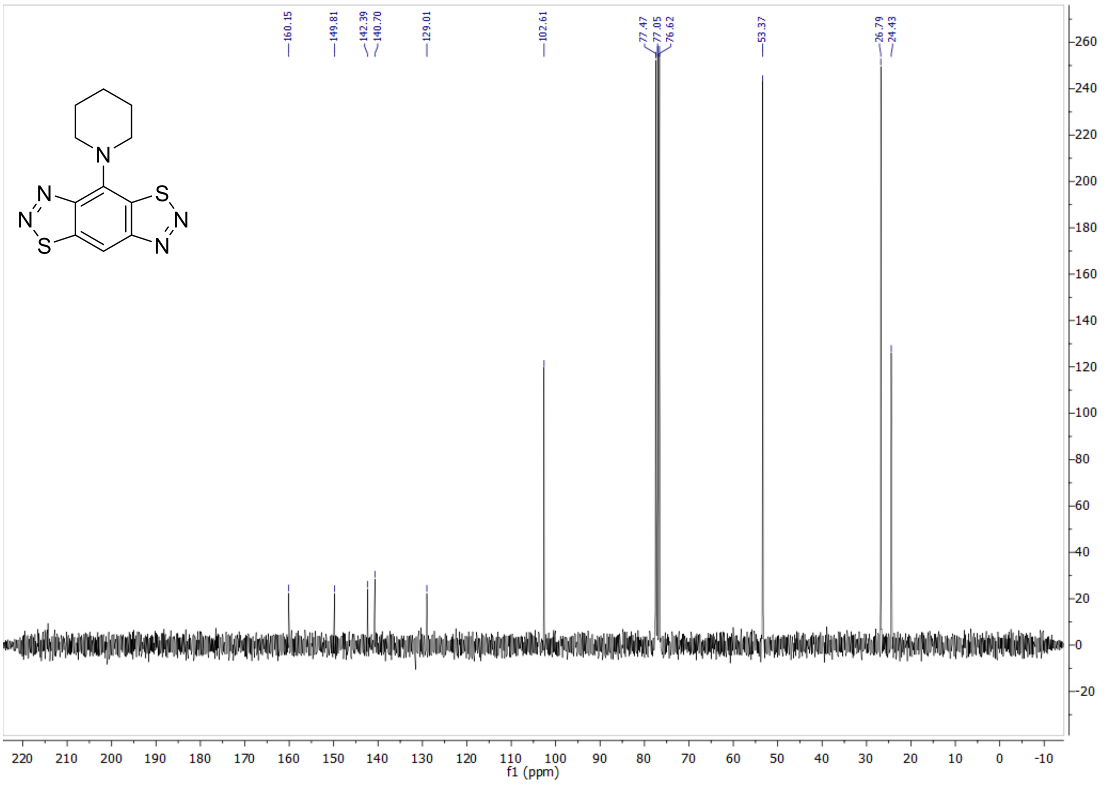

4-(Pyrrolidin-1-yl)benzo[1,2-d:4,5-d']bis([1,2,3]thiadiazole) (4c)

^{1}H NMR (300 MHz)

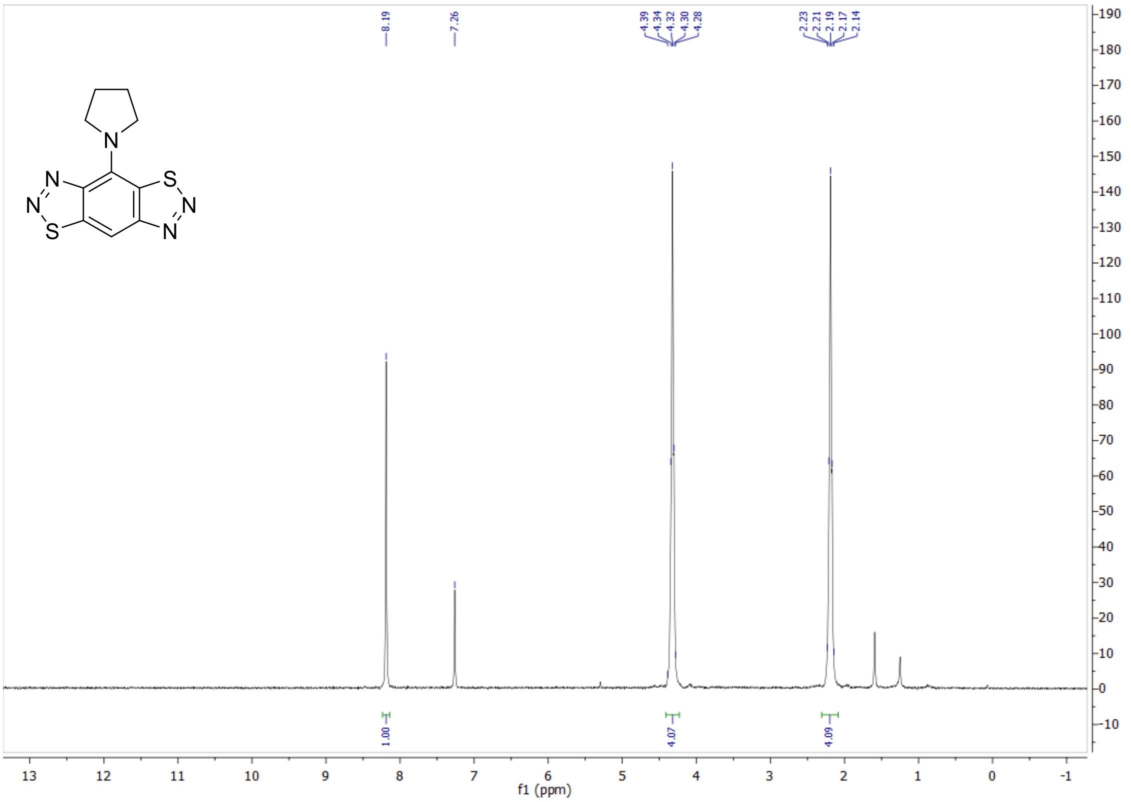

^{13}C NMR(75 MHz)

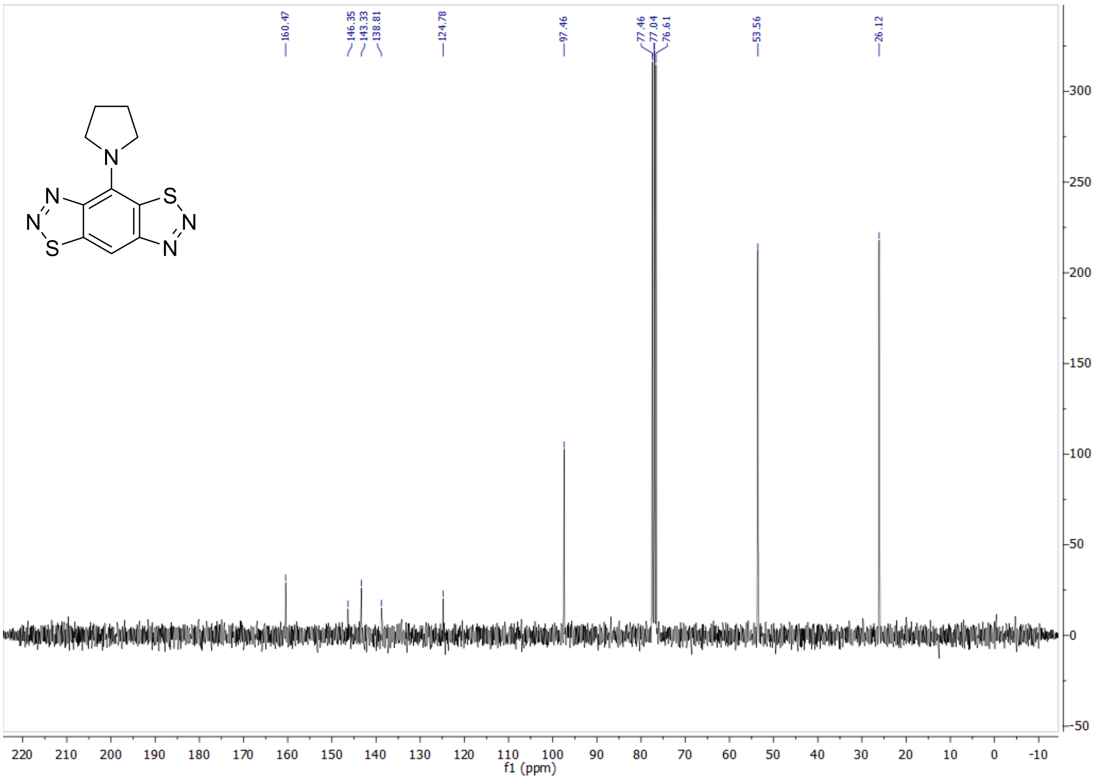

N-phenylbenzo[1,2-d:4,5-d']bis([1,2,3]thiadiazole)-4-amine (4e)

^{1}H NMR (300 MHz)

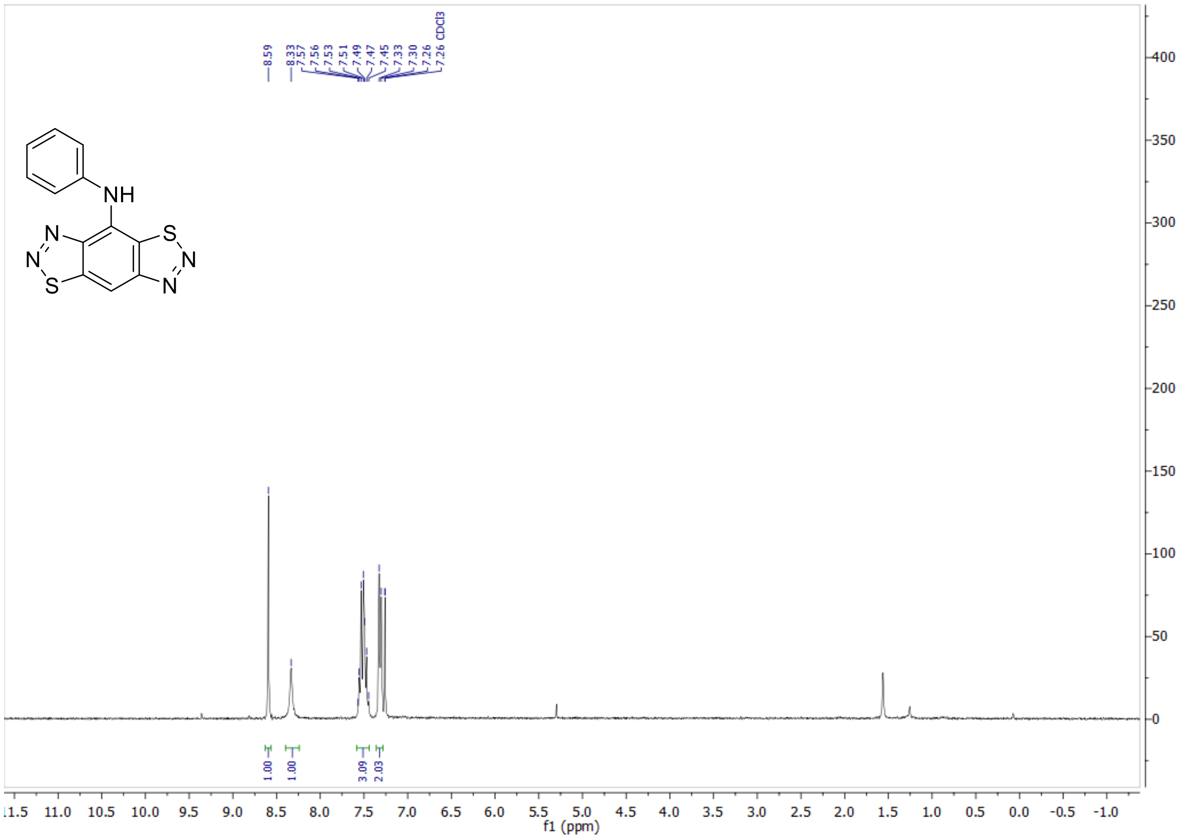

^{13}C NMR(75 MHz)

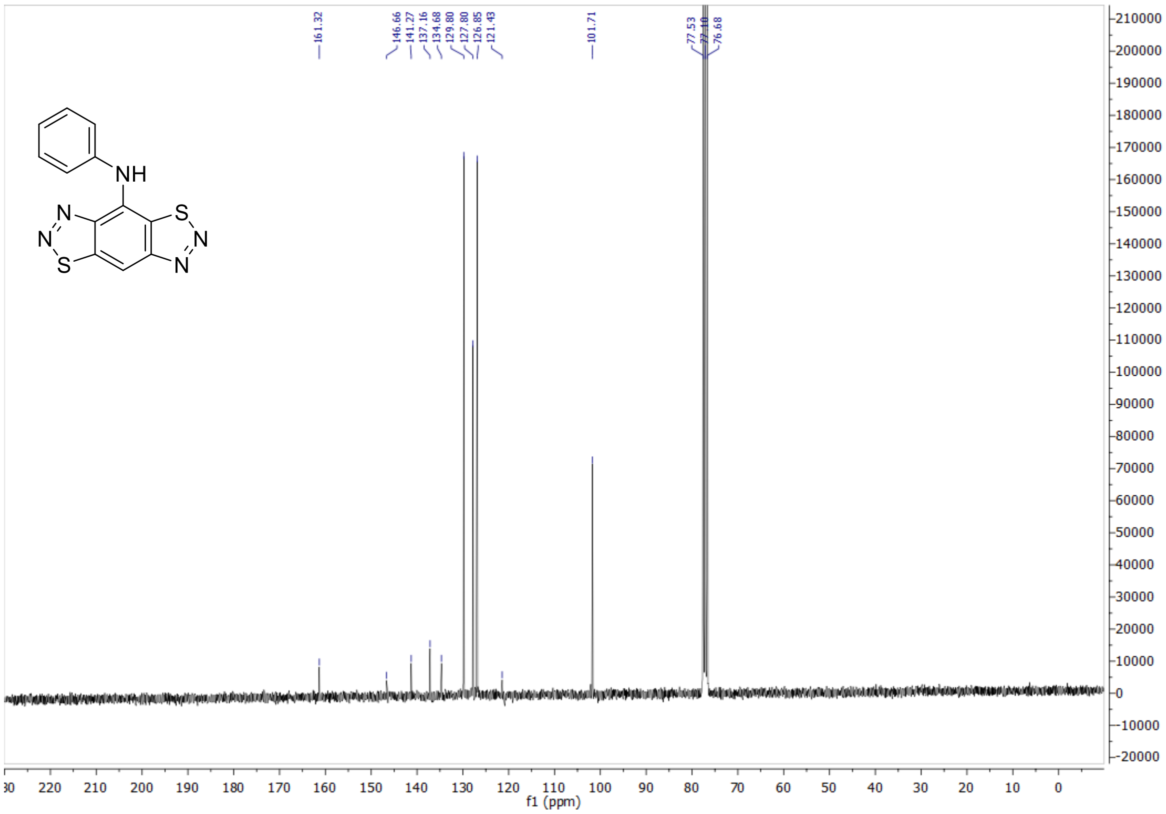

4-(Phenylthio)benzo[1,2-d:4,5-d']bis([1,2,3]thiadiazole) (6a)

^{1}H NMR (300 MHz)

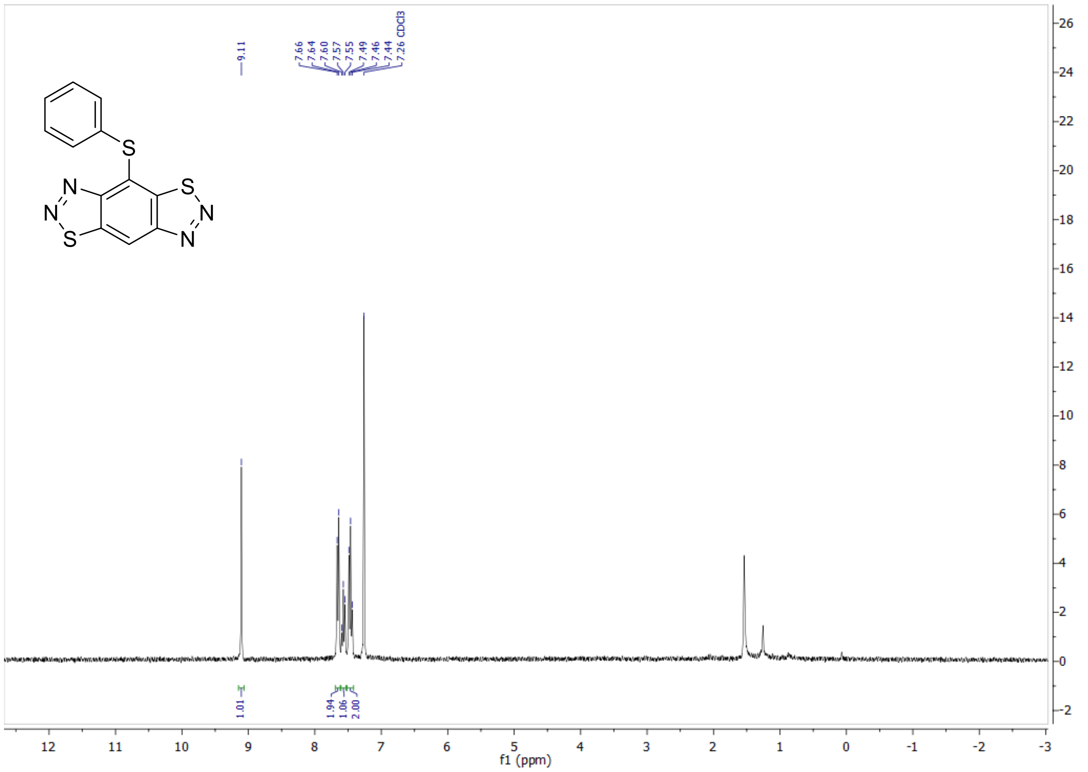

^{13}C NMR(75 MHz)

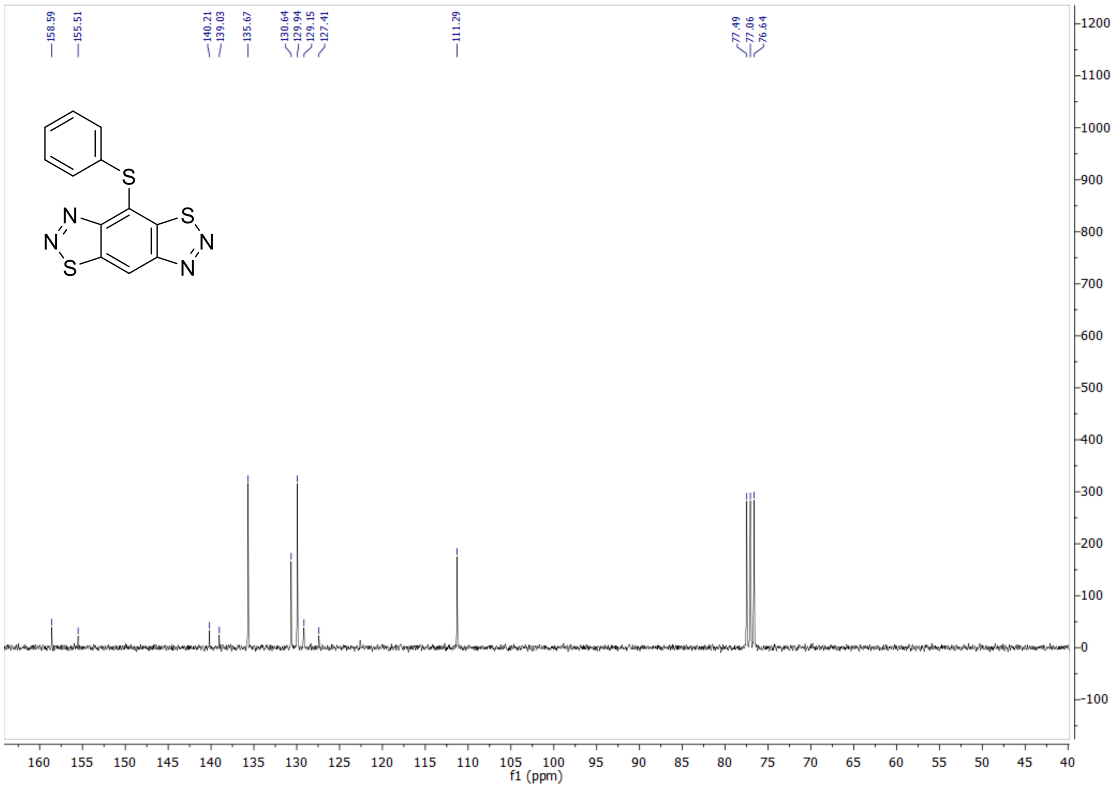

4-(Hexylthio)benzo[1,2-d:4,5-d']bis([1,2,3]thiadiazole) (6b)

^{1}H NMR (300 MHz)

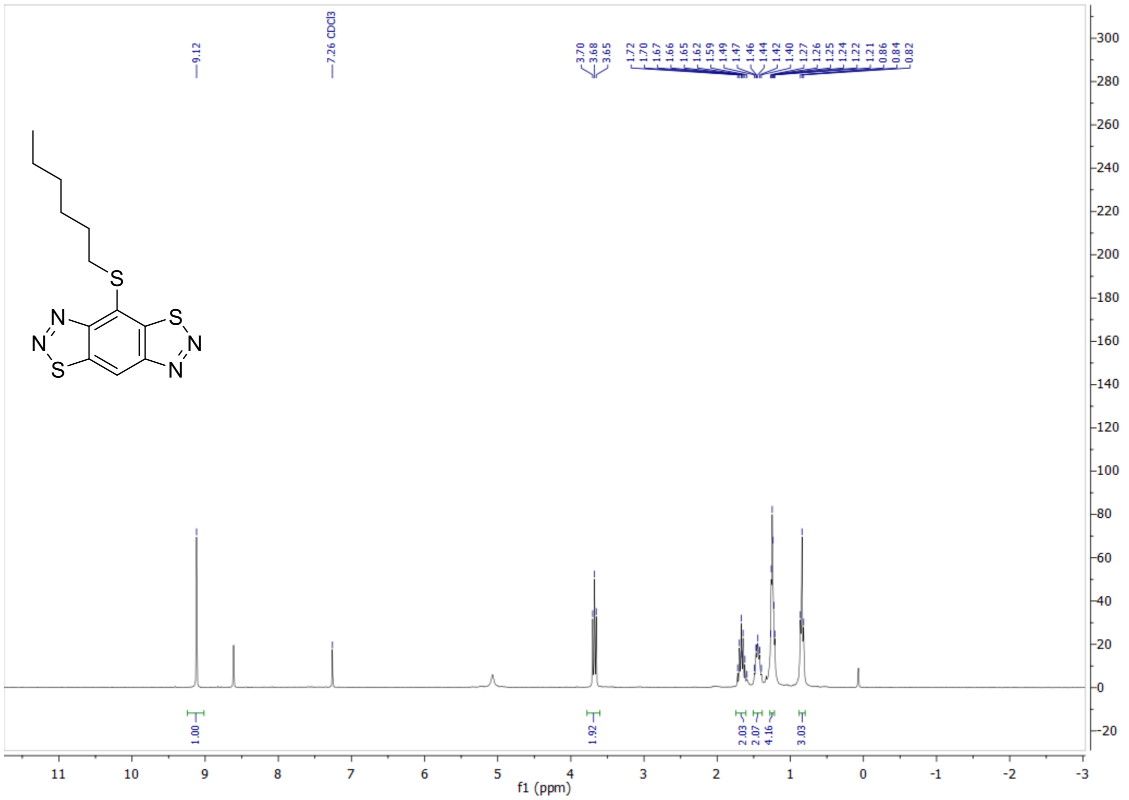

^{13}C NMR(75 MHz)

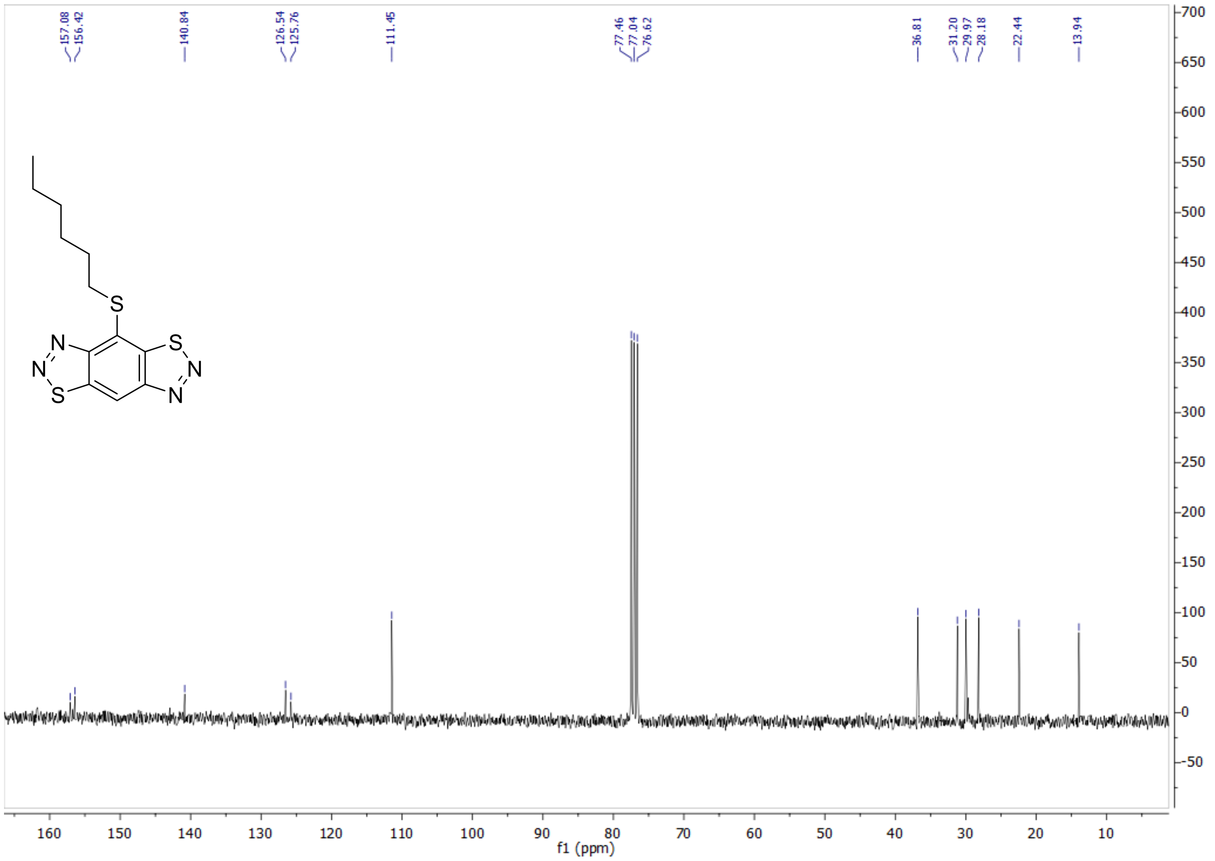

4-(Dodecylthio)benzo[1,2-d:4,5-d']bis([1,2,3]thiadiazole) (6c)

^{1}H NMR (300 MHz)

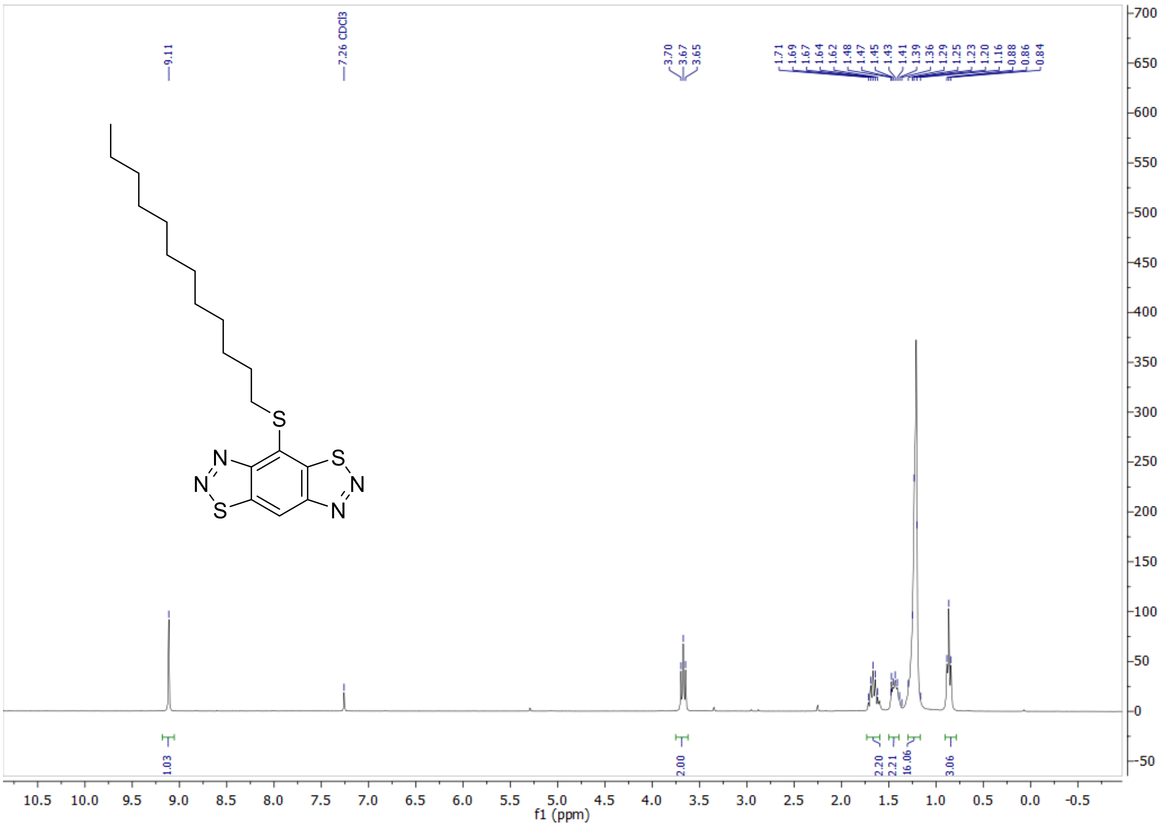

^{13}C NMR(75 MHz)

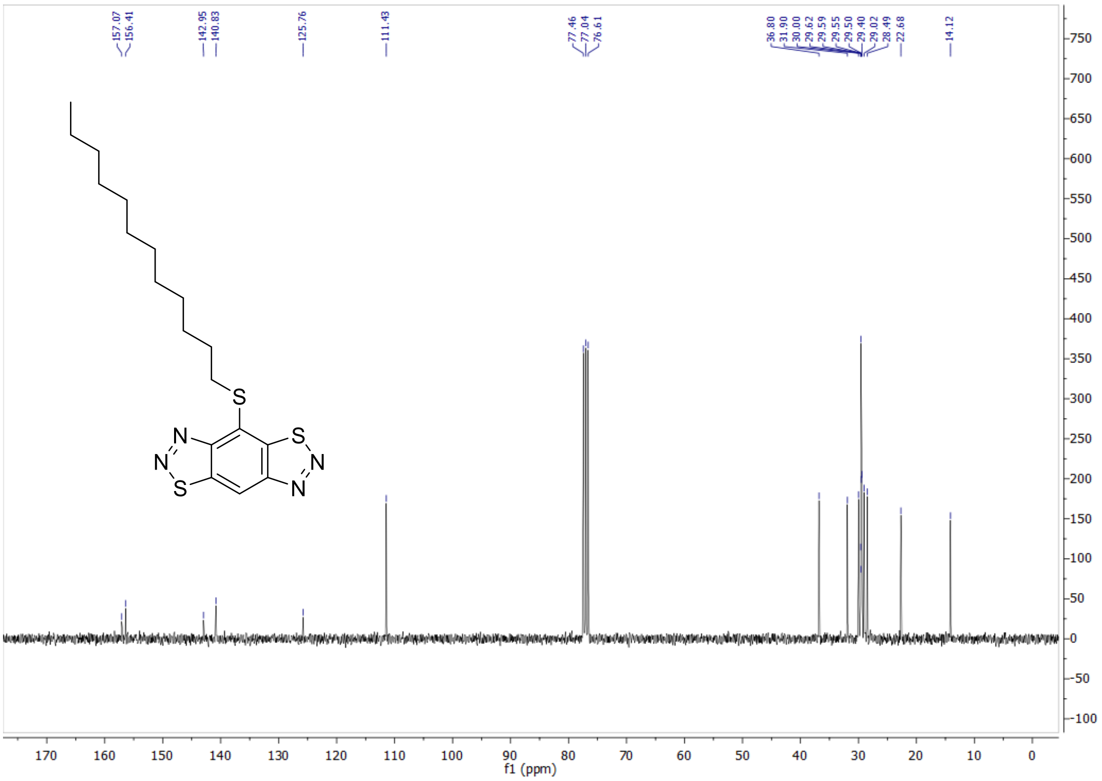

4-(Thiophen-2-yl)benzo[1,2-d:4,5-d']bis([1,2,3]thiadiazole) (8a)

^{1}H NMR (300 MHz)

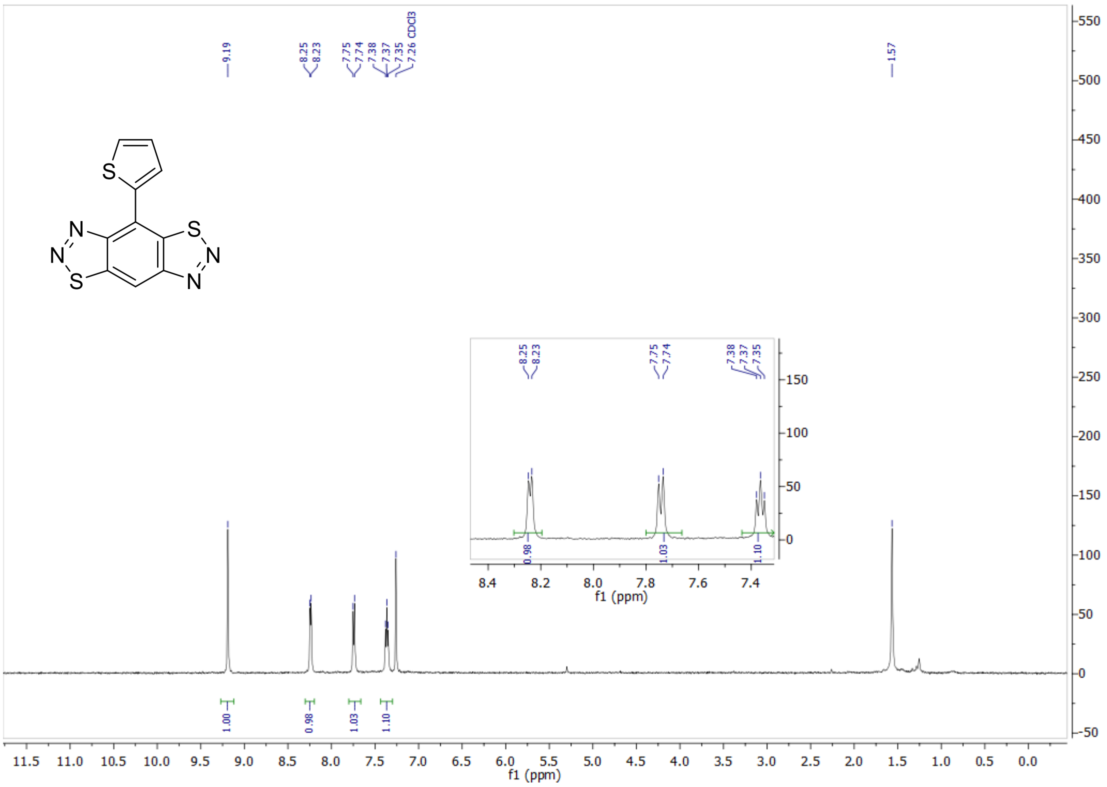

^{13}C NMR(75 MHz)

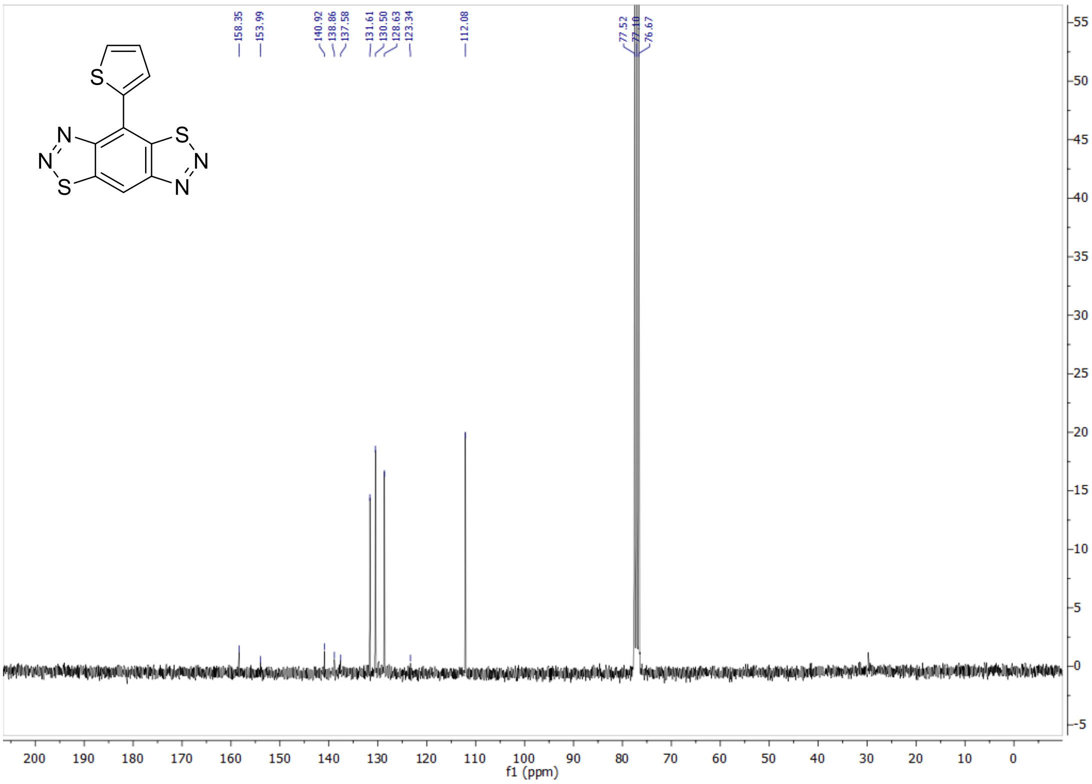

4-(4-Hexylthiophen-2-yl)benzo[1,2-d:4,5-d']bis([1,2,3]thiadiazole) (8b)

^{1}H NMR (300 MHz)

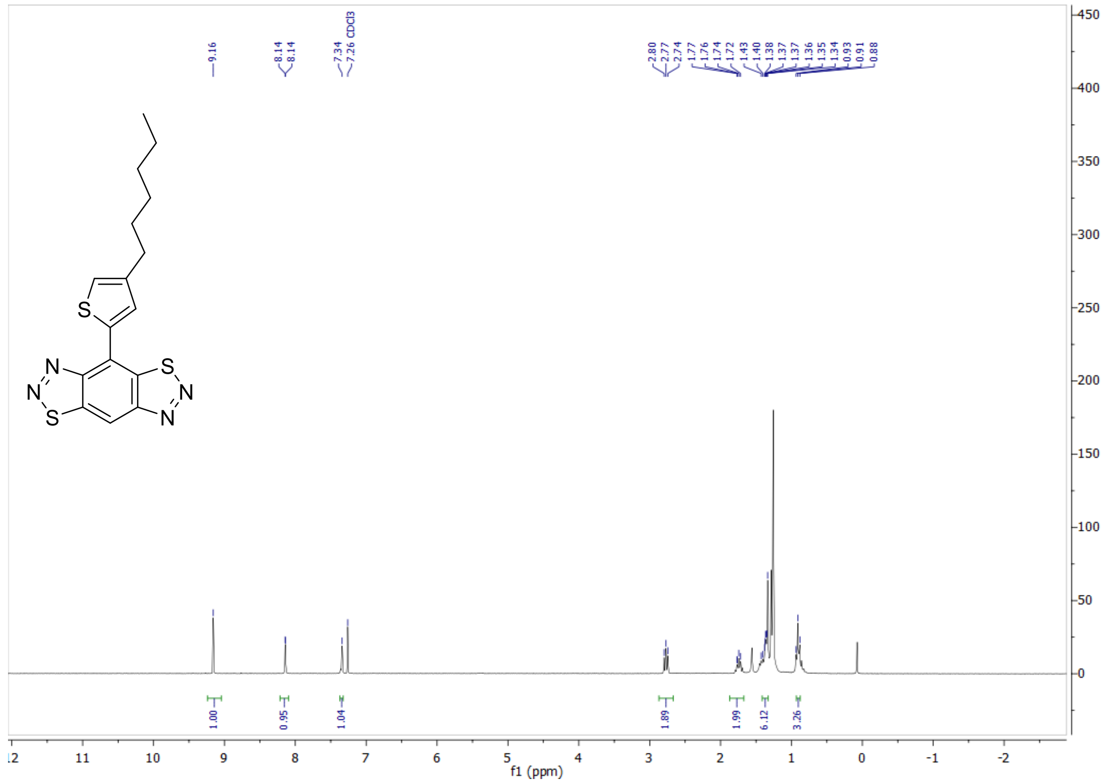

^{13}C NMR(75 MHz)

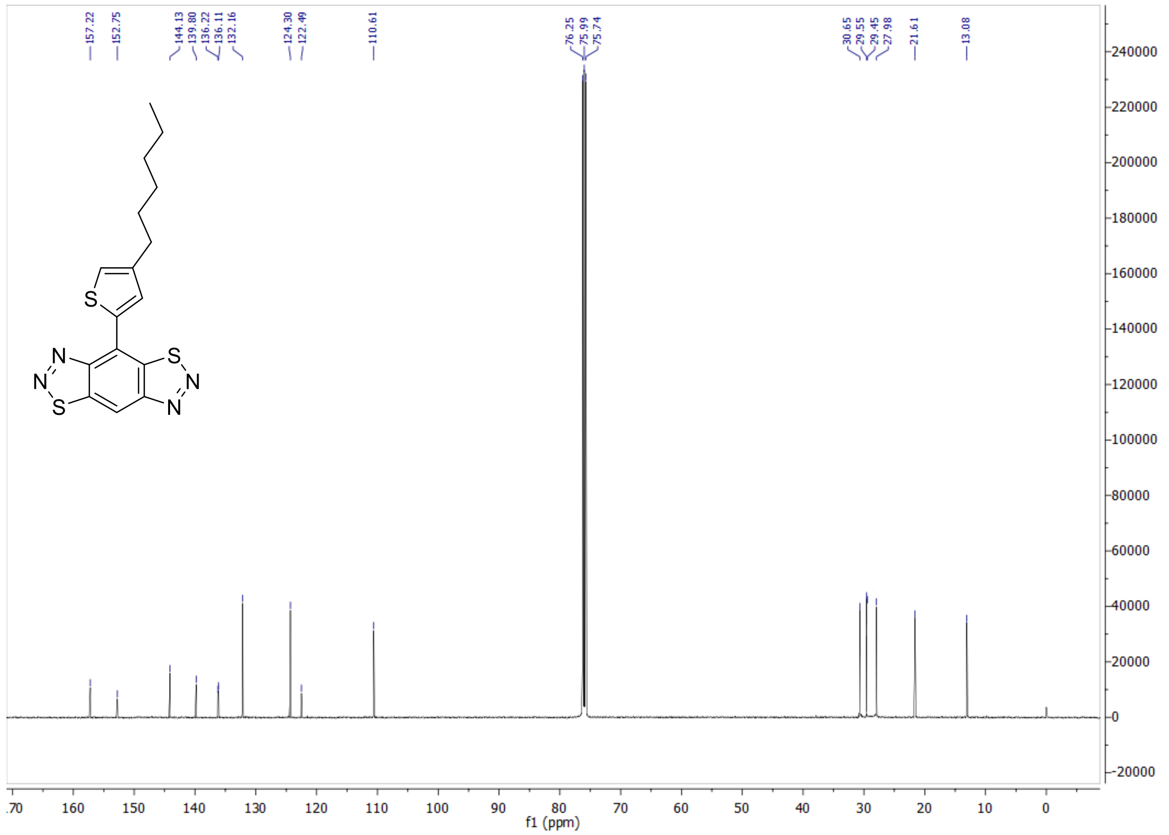

4-(5'-(Trimethylsilyl)-[2,2'-bithiophen]-5-yl)benzo[1,2-*d*:4,5-*d*']bis([1,2,3]thiadiazole) (8c)

^{1}H NMR (300 MHz)

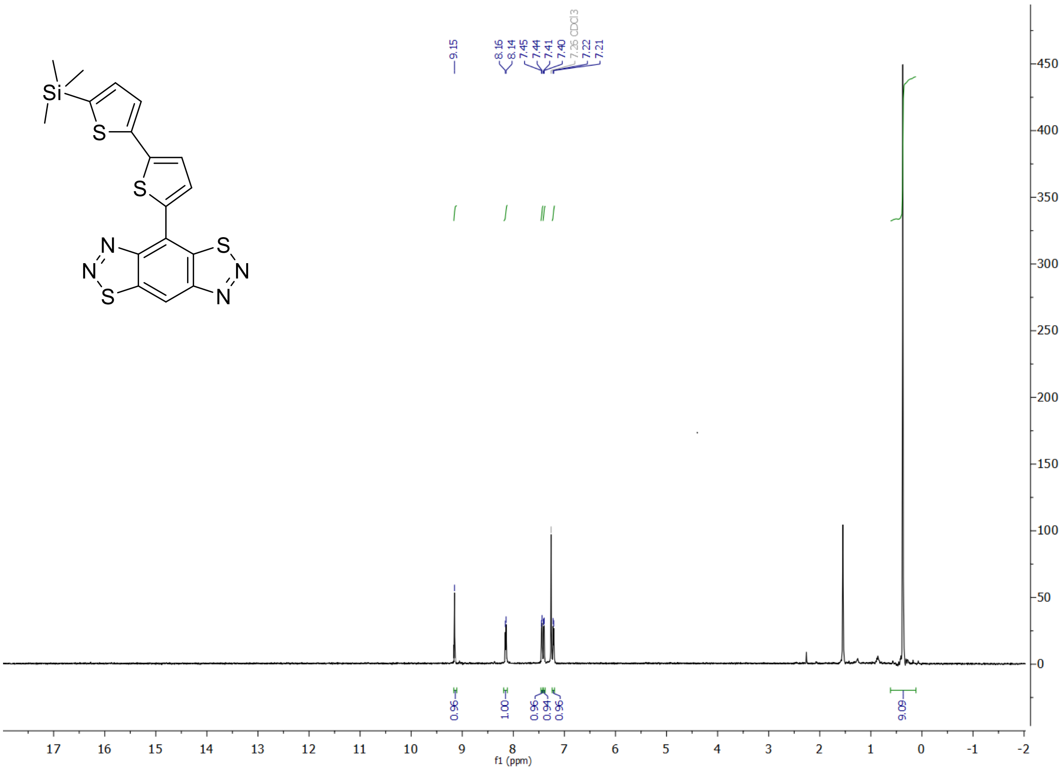

^{13}C NMR(75 MHz)

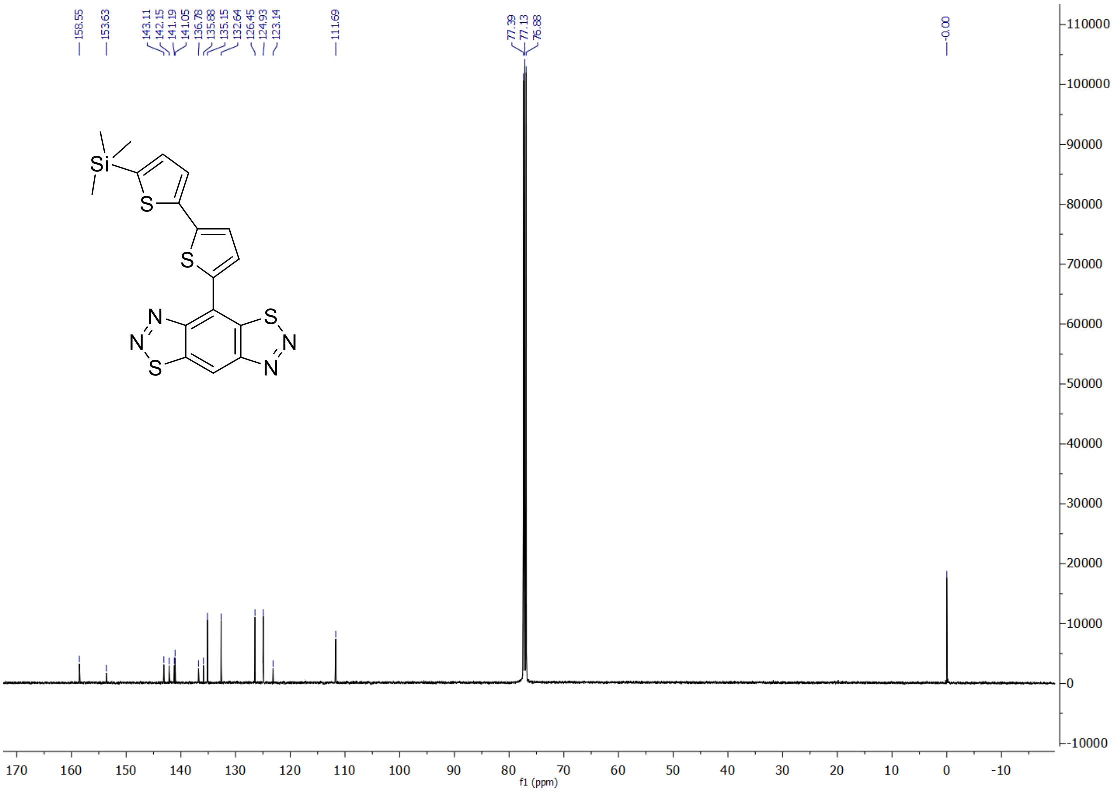

4-(5-(2-Ethylhexyl)thiophen-2-yl)benzo[1,2-*d*:4,5-*d*']bis([1,2,3]thiadiazole) (8d)

^{1}H NMR (300 MHz)

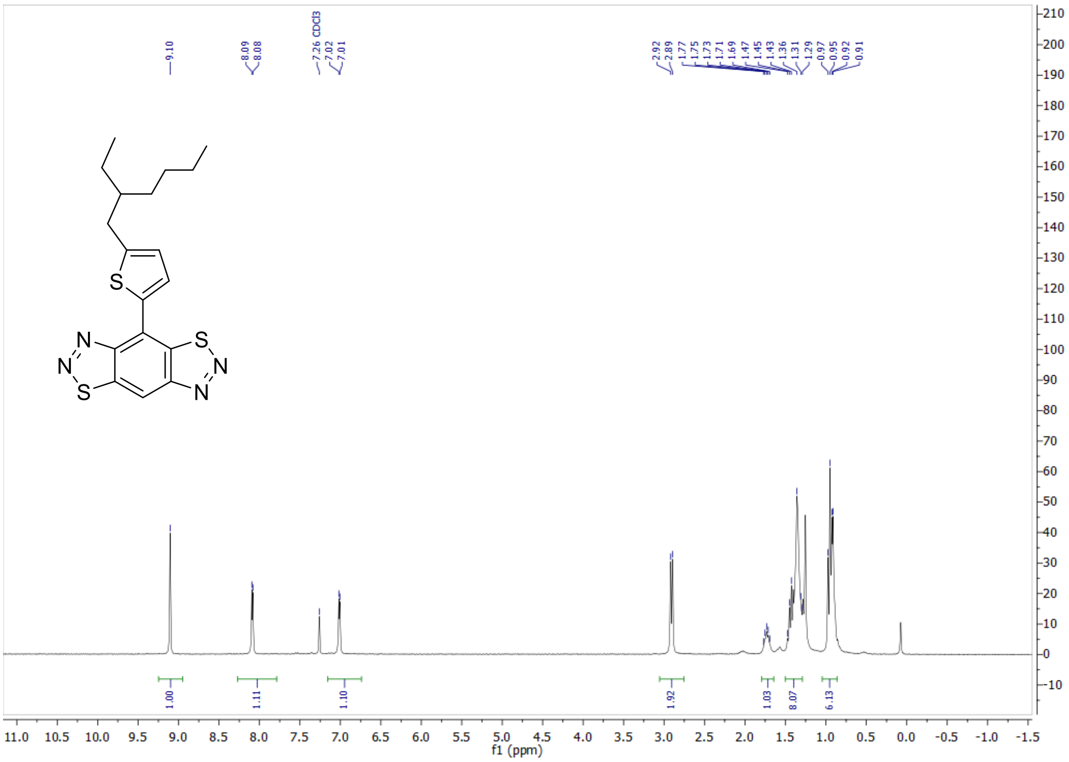

^{13}C NMR(75 MHz)

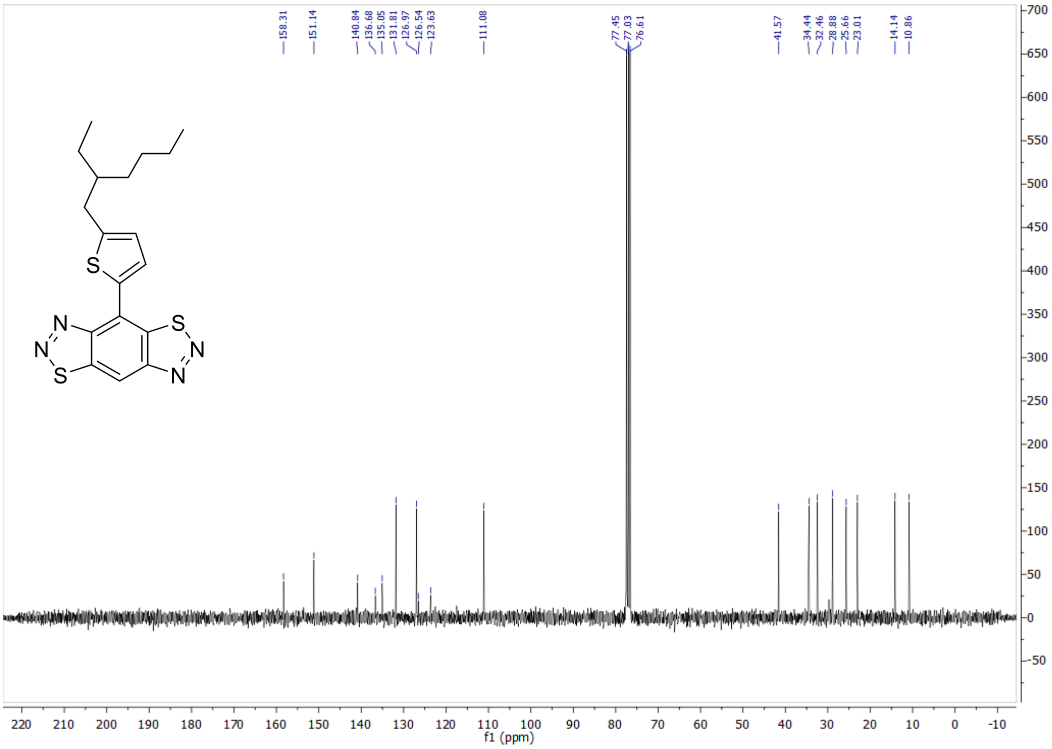

4-Phenylbenzo[1,2-*d*:4,5-*d*']bis([1,2,3]thiadiazole) (8e)

^{1}H NMR (300 MHz)

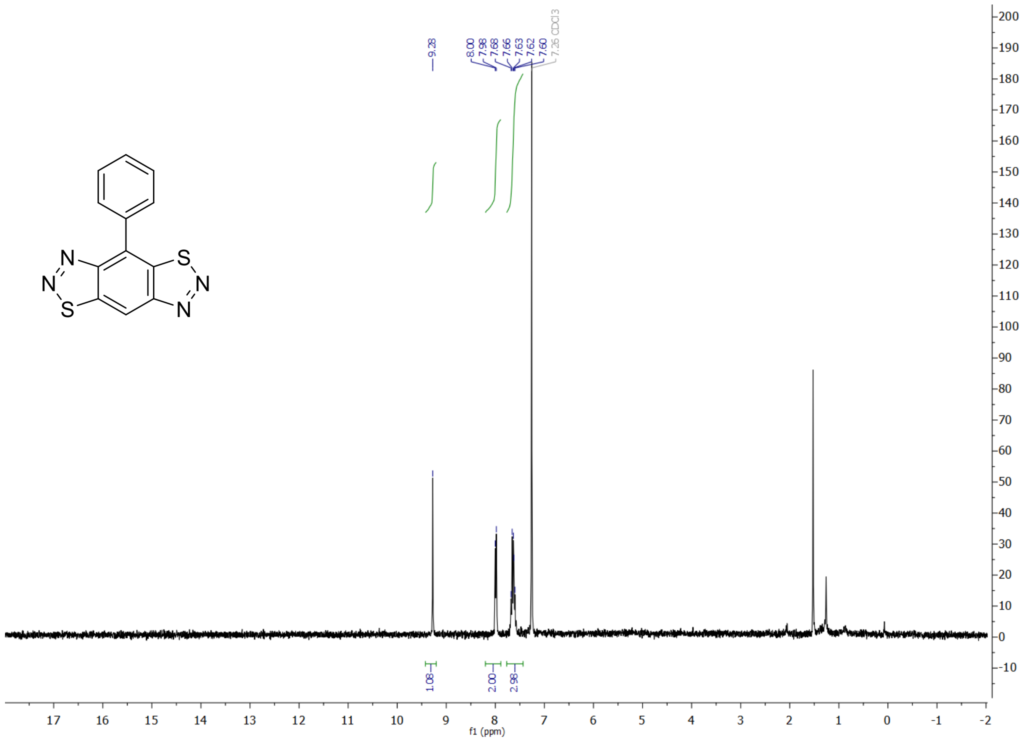

^{13}C NMR(75 MHz)

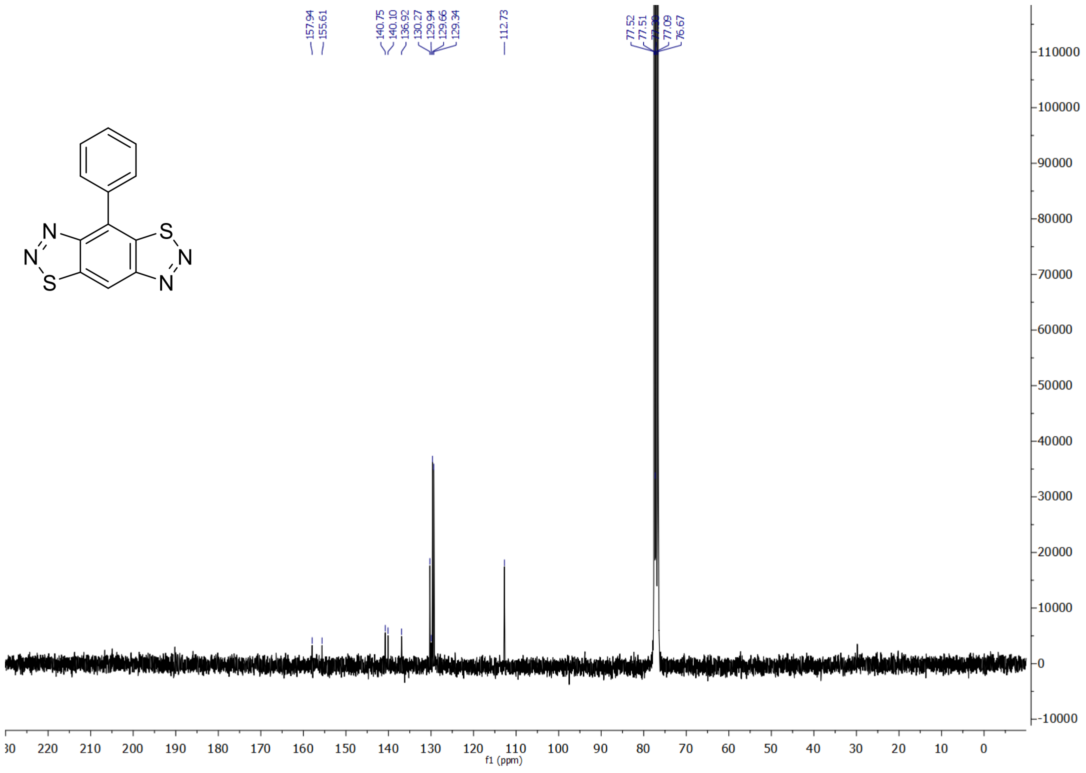

4-(p-Tolyl)benzo[1,2-d:4,5-d']bis([1,2,3]thiadiazole) (8f)

^{1}H NMR (300 MHz)

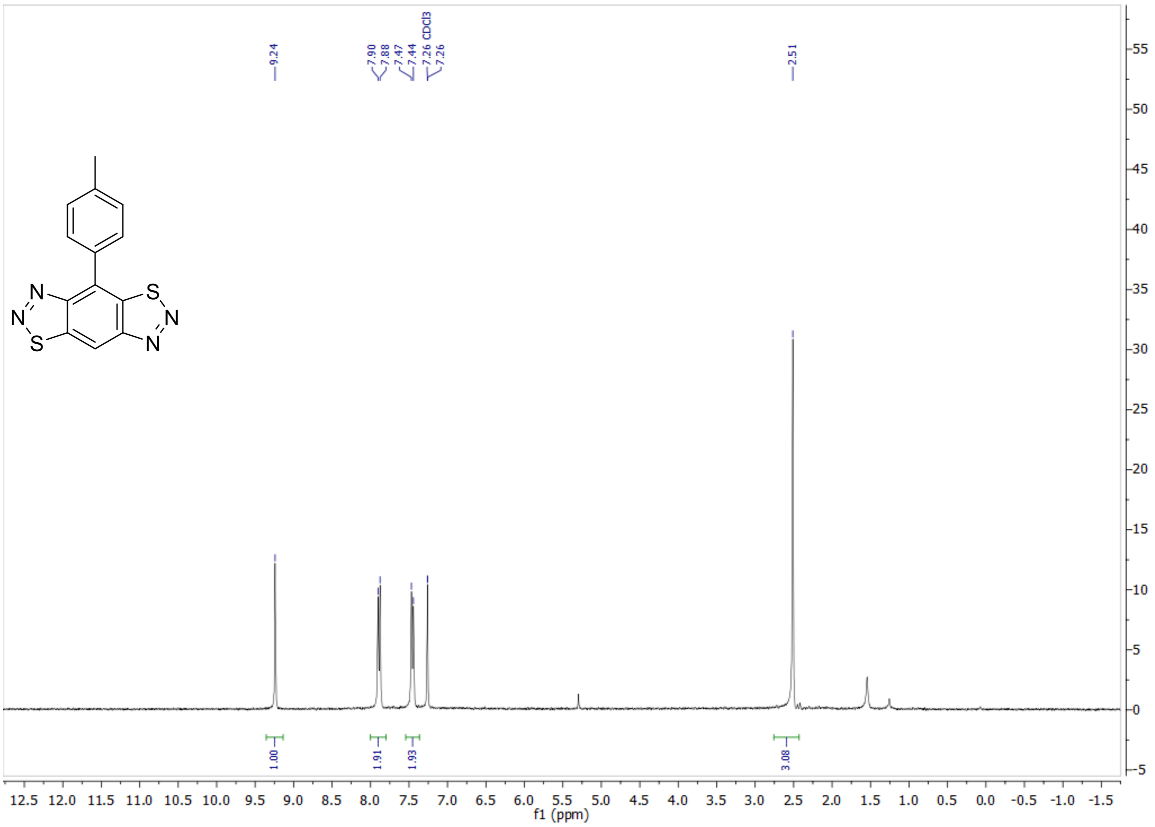

^{13}C NMR(75 MHz)

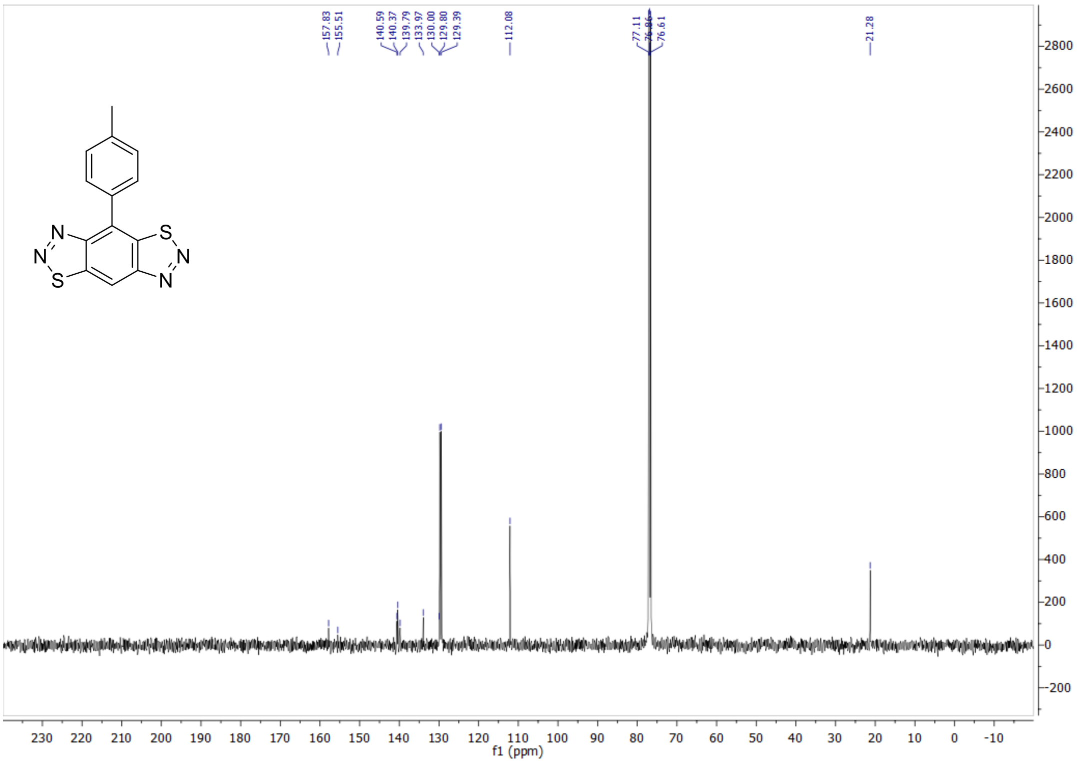

4-(4-Methoxyphenyl)benzo[1,2-*d*:4,5-*d*']bis([1,2,3]thiadiazole) (8g)

^{1}H NMR (300 MHz)

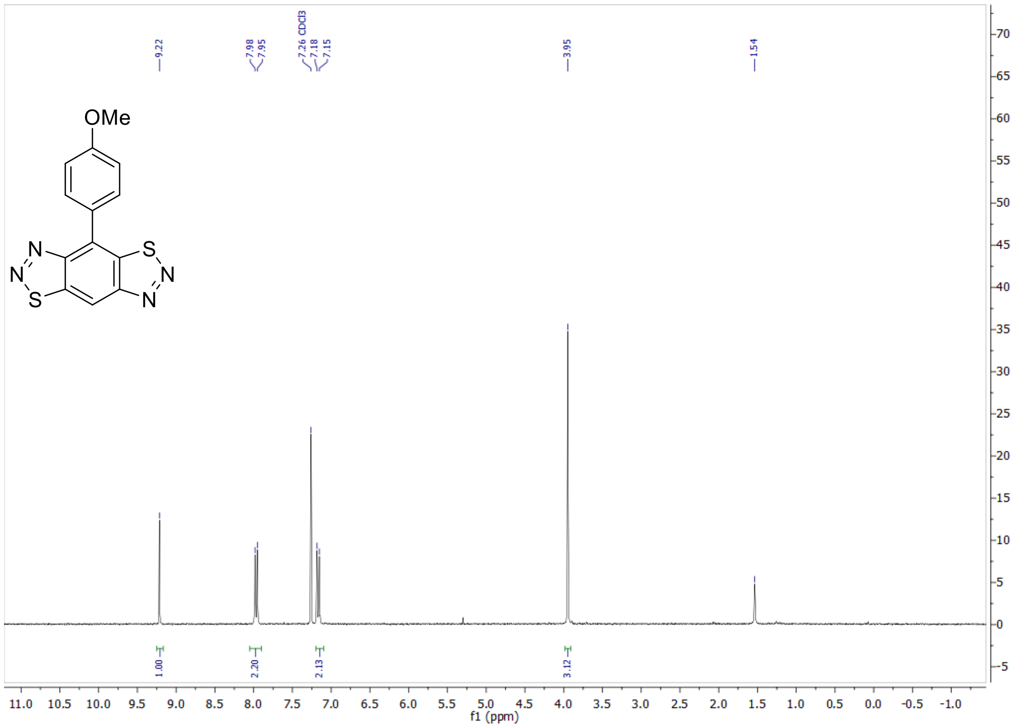

^{13}C NMR(75 MHz)

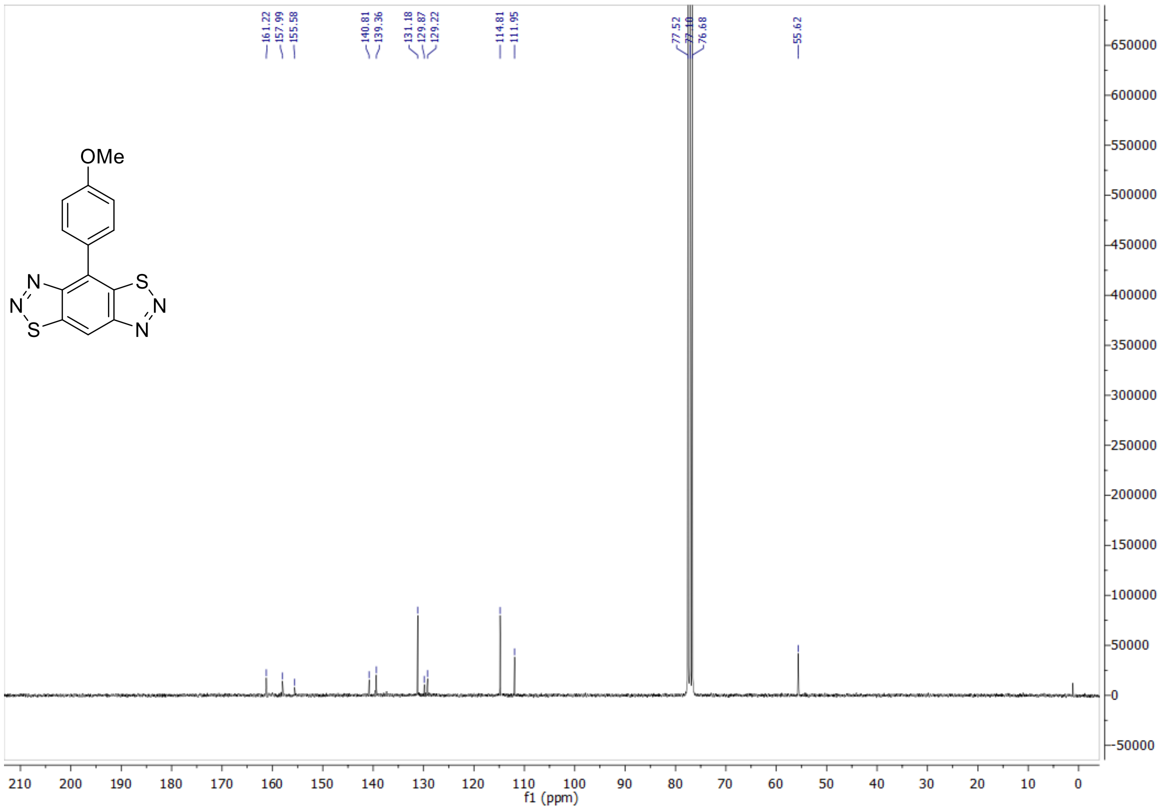

4-(Benzo[1,2-*d*:4,5-*d*']bis([1,2,3]thiadiazole)-4-yl)-*N*,*N*-diphenylaniline (8h)

^{1}H NMR (300 MHz)

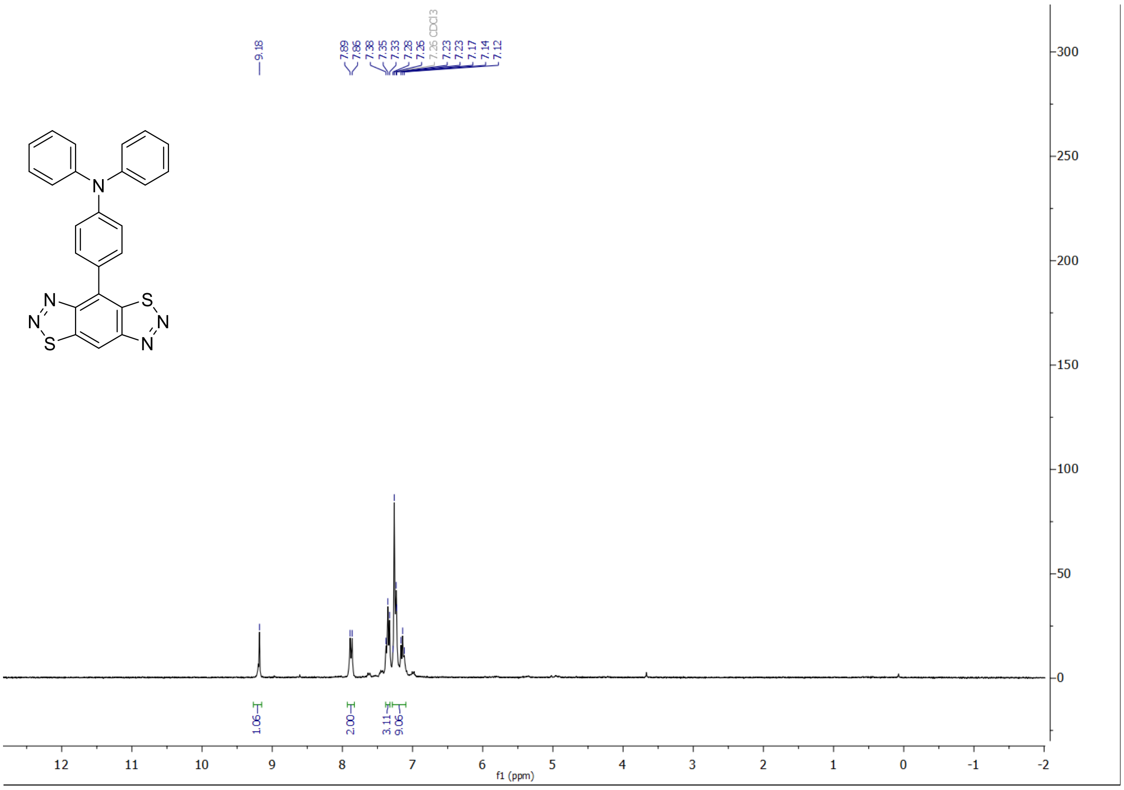

^{13}C NMR(75 MHz)

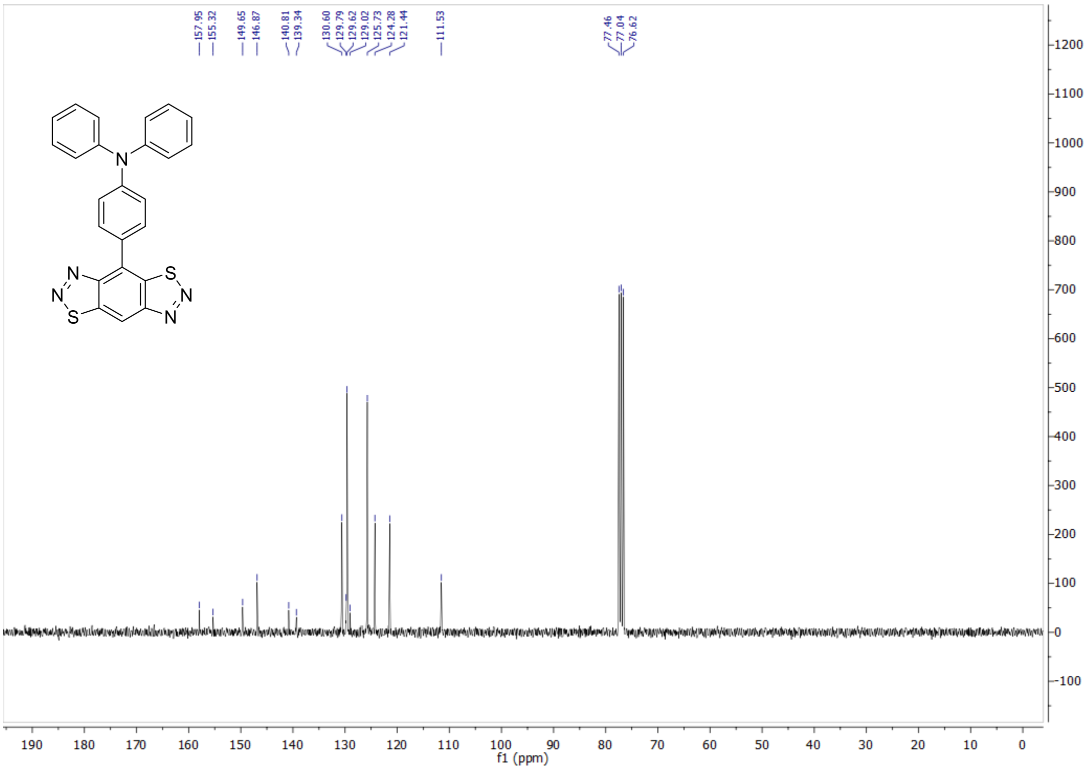

Table S4. Crystallographic data for **1- 3**

Compound	**1**	**2**	**3**
Formula moiety	$_2(C_3HN_2S)$	$C_6HBrN_4S_2$	$C_6Br_2N_4S_2$
Brutto formula	$C_6H_2N_4S_2$	$C_6HBrN_4S_2$	$C_6Br_2N_4S_2$
Formula weight	194.24	273.14	352.04
Diffractometer	Bruker QUEST	XtaLAB Synergy	XtaLAB Synergy
Scan mode	ω and ϕ scans	ω-scans	ω-scans
Anode [Wavelength, Å]	MoKα [0.71073] microfocus sealed X-ray tube	Mo Kα [0.71073] micro-focus sealed X-ray tube	Mo Kα [0.71073] micro-focus sealed X-ray tube
Crystal Dimensions, mm	0.11 × 0.32 × 0.39	0.05 × 0.05 × 0.2	0.2 × 0.3 × 0.56
Crystal color	yellow	yellow	clear yellow
Crystal system	triclinic	monoclinic	monoclinic
a, Å	3.738(3)	12.5976(3)	16.1779(4)
b, Å	9.027(5)	9.2941(2)	3.88730(10)
c, Å	10.156(9)	6.9912(2)	16.1804(4)
α, °	82.88(4)	90	90
β, °	87.48(4)	103.562(3)	119.312(3)
γ, °	83.96(4)	90	90
Volume, $Å^3$	338.0(4)	795.73(4)	887.28(4)
Density, gcm^{-3}	1.909	2.280	2.635
Temperature, K	100	99.97(18)	100.00(10)
T_{min}/T_{max}	0.480154/0.746072	0.55126/1.00000	0.12670/1.00000
μ, mm^{-1}	0.718	5.636	9.562
Space group	P-1	$P2_1/c$	$P2_1/n$

Z	2	4	4
F(000)	196	528	664
Reflections collected	2024	19591	28713
Independent reflections	2024	3403	1924
Reflections (I>2σ(I))	1881	2983	1906
Parameters	110	122	128
R_{int}	0.00	0.0366	0.0815
$2\theta_{min}$ - $2\theta_{max}$, °	4.044 - 61.254	5.502 - 71.638	4.984 - 53.992
wR_2 (all reflections)	0.0881	0.0675	0.1057
R_1(I>σ(I))	0.0361	0.0286	0.0390
GOF	1.092	1.038	1.061
ρ_{min}/ρ_{max}, eÅ^{-3}	-0.393/0.515	-0.520/0.874	-0.660/2.192

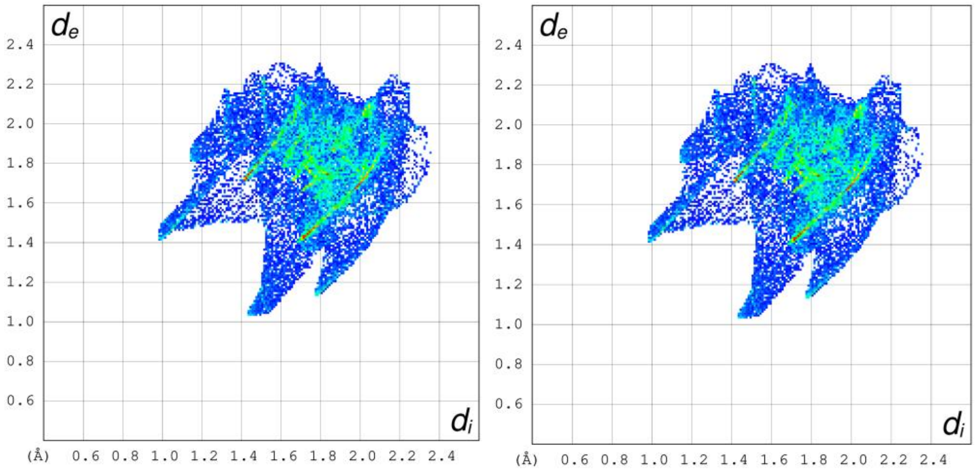

Figure S2. 2D-fingerprint plots for two crystallographically independent molecules in crystal structure of **1**.

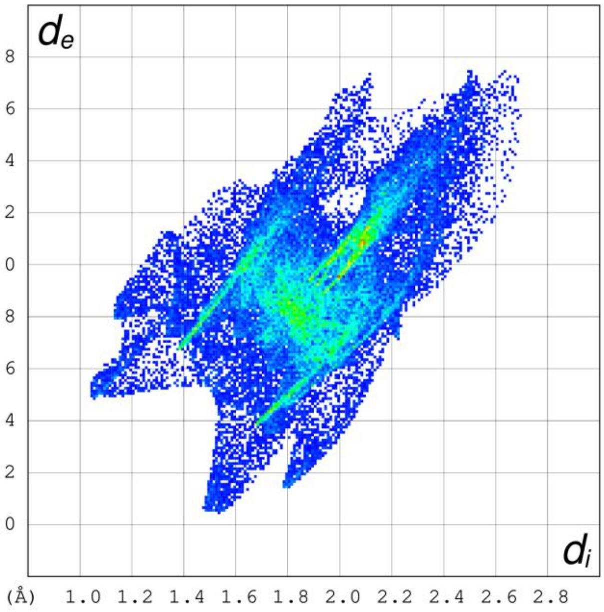

Figure S3. 2D-fingerprint plot for **2**.

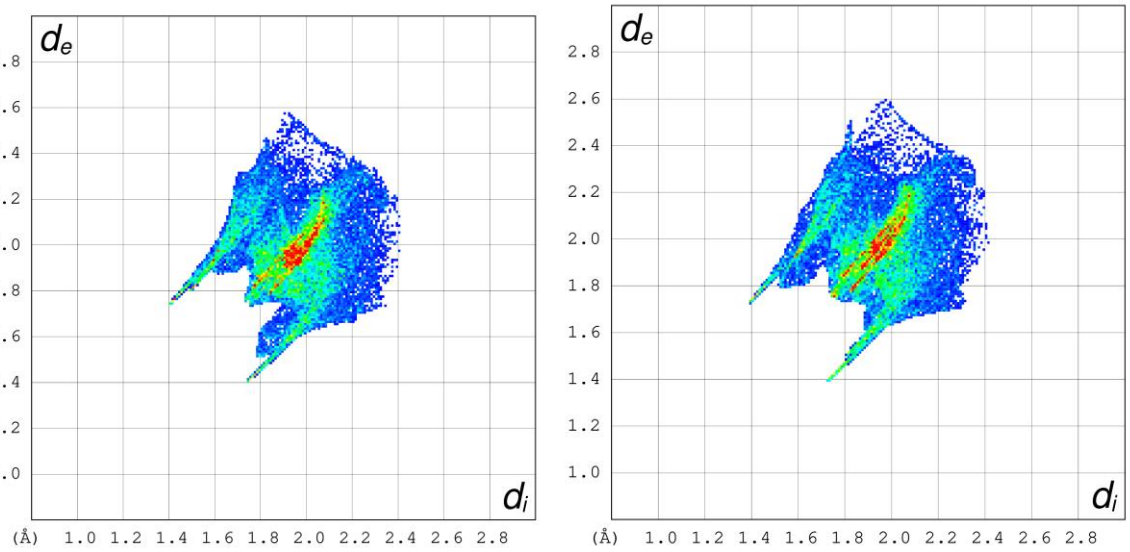

Figure S4. 2D-fingerprint plots for two crystallographically independent molecules in crystal structure of **3**.
